# Desiccation Tolerance in *Ramonda serbica* Panc.: An Integrative Transcriptomic, Proteomic, Metabolite and Photosynthetic Study

**DOI:** 10.3390/plants11091199

**Published:** 2022-04-28

**Authors:** Marija Vidović, Ilaria Battisti, Ana Pantelić, Filis Morina, Giorgio Arrigoni, Antonio Masi, Sonja Veljović Jovanović

**Affiliations:** 1 Institute of Molecular Genetics and Genetic Engineering, Laboratory for Plant Molecular Biology, University of Belgrade, Vojvode Stepe 444a, 11042 Belgrade, Serbia; anapantelic@imgge.bg.ac.rs; 2 Department of Biomedical Sciences, University of Padova, Via Ugo Bassi 58/B, 35131 Padova, Italy; ilaria.battisti@studenti.unipd.it (I.B.); giorgio.arrigoni@unipd.it (G.A.); 3 Proteomics Center, University of Padova and Azienda Ospedaliera di Padova, Via G. Orus 2/B, 35129 Padova, Italy; 4 Biology Center of the Czech Academy of Sciences, Institute of Plant Molecular Biology, Department of Plant Biophysics and Biochemistry, Branišovska 31/1160, 370 05 Ceske Budejovice, Czech Republic; morina@umbr.cas.cz; 5 Department of Agronomy, Food, Natural Resources, Animals and Environment, University of Padova, Viale dell’Università 16, 35020 Legnaro, Italy; antonio.masi@unipd.it; 6 Institute for Multidisciplinary Research, Department of Life Science, University of Belgrade, Kneza Viseslava 1, 11000 Belgrade, Serbia

**Keywords:** cell wall remodeling, cyclic electron transport, drought, germin-like proteins, late embryogenesis abundant proteins, OJIP, pectin, polyphenol oxidase, resurrection plant, superoxide dismutase

## Abstract

The resurrection plant *Ramonda serbica* Panc. survives long desiccation periods and fully recovers metabolic functions within one day upon watering. This study aimed to identify key candidates and pathways involved in desiccation tolerance in *R. serbica.* We combined differential transcriptomics and proteomics, phenolic and sugar analysis, FTIR analysis of the cell wall polymers, and detailed analysis of the photosynthetic electron transport (PET) chain. The proteomic analysis allowed the relative quantification of 1192 different protein groups, of which 408 were differentially abundant between hydrated (HL) and desiccated leaves (DL). Almost all differentially abundant proteins related to photosynthetic processes were less abundant, while chlorophyll fluorescence measurements implied shifting from linear PET to cyclic electron transport (CET). The levels of H_2_O_2_ scavenging enzymes, ascorbate-glutathione cycle components, catalases, peroxiredoxins, Fe-, and Mn superoxide dismutase (SOD) were reduced in DL. However, six germin-like proteins (GLPs), four Cu/ZnSOD isoforms, three polyphenol oxidases, and 22 late embryogenesis abundant proteins (LEAPs; mainly LEA4 and dehydrins), were desiccation-inducible. Desiccation provoked cell wall remodeling related to GLP-derived H_2_O_2_/HO^●^ activity and pectin demethylesterification. This comprehensive study contributes to understanding the role and regulation of the main metabolic pathways during desiccation aiming at crop drought tolerance improvement.

## 1. Introduction

Drought is a major cause of massive economic losses in agriculture. It affects more than 60% of the global land area [1]. Current approaches aimed at improving crop drought tolerance, besides classical breeding, are focused on various biotechnological and synthetic biology studies based on the transfer of drought-tolerant traits. The success of these approaches depends on our understanding of the molecular mechanisms underlying drought endurance [2].

Drought-tolerant higher plants can tolerate up to 60% water loss [3]. Vascular plant species able to survive desiccation (loss of 90–98% of water content) for months without permanent damage to leaves and roots are rare, and they are known as resurrection plants [4]. Resurrection plants can fully resume metabolic functions upon rewatering in a very short period, even within 24 h [5]. Thus, resurrection plants have emerged as model organisms to study plant desiccation tolerance with potential application in improving drought tolerance in crops [6,7,8].

At the cellular level, dehydration induces protein denaturation and aggregation and alters the structural organization of membrane lipids, resulting in altered fluidity and loss of membrane integrity [2]. Besides osmotic stress, desiccation induces the excessive accumulation of reactive oxygen species (ROS), e.g., the superoxide anion radical (O_2_^•–^), hydrogen peroxide (H_2_O_2_), and the most toxic hydroxyl radical (HO^•^) [9].

Plants adapt to drought by several physiological and morphological mechanisms [10]. Resurrection plants have evolved unique desiccation tolerance strategies, including the upregulation of genes encoding protective proteins, such as aldehyde dehydrogenases, heat shock and late embryogenesis abundant proteins (LEAPs) [11,12]. In addition, genes associated with cell wall remodeling and the accumulation of osmotically active compounds, phenolics, and antioxidative enzymes are upregulated under desiccation [6]. Furthermore, downregulation of photosynthesis in resurrection species serves to minimize ROS production during desiccation [13]. In poikilochlorophyllous resurrection plants, characterized by the loss of chlorophyll and integrity of thylakoid membranes upon desiccation, it might be expected that generation of ROS related to photosynthetic electron transport (PET) is disabled. In homoiochlorophyllous resurrection plants, such as *Craterostigma plantagineum* and *Haberlea rhodopensis*, photosynthesis is reversibly inactivated, since they retain their chlorophyll and thylakoid structure enabling them to recover rapidly upon rehydration [11,14]. Therefore, protective mechanisms, such as leaf folding to reduce absorbed radiation or/and accumulation of antioxidants, anthocyanins, and other phenolic compounds against solar radiation should minimize oxidative stress [8].

An accumulation of protective LEAPs, which may stabilize the correct structure of proteins and membranes during cellular dehydration is considered a hallmark of desiccation tolerance in various organisms (e.g., bacteria, rotifers, nematodes, brine shrimps), not only in plants [15]. Although no specific physiological function was attributed to LEAPs [16], their high structural plasticity allows them to interact with various ligands and partners.

*Ramonda serbica* Panč. is a typical homoiochlorophyllous resurrection plant [17]. It belongs to the Gesneriaceae family that encompasses other resurrection species (*H. rhodopensis, Boea hydrometrica*) extensively described in the literature [7,18]. To date, no differential transcriptomic or proteomic studies have been performed on *R. serbica*. From the evolutionary aspect, as an endemic and tertiary relict, *R. serbica* is an excellent model to study vegetative desiccation tolerance, a phenomenon that is considered a critical step in the evolution of primitive land plants [19].

This study aimed to identify key candidates and pathways involved in the desiccation tolerance mechanisms in *R. serbica.* Desiccation tolerance is regulated by a complex network of genes and proteins. To achieve this, a systems biology approach, combining transcriptomics, proteomics, and analysis of specific metabolites was employed. Differential gene expression analysis and TMT-based comparative quantitative proteomics of hydrated and desiccated leaves were correlated with photosynthetic performance, structural properties of the cell wall, enzyme activities, and metabolic pathways.

## 2. Results

### 2.1. Changes in the Transcriptome and Proteome of R. serbica HL and DL 

**Figure 1:**
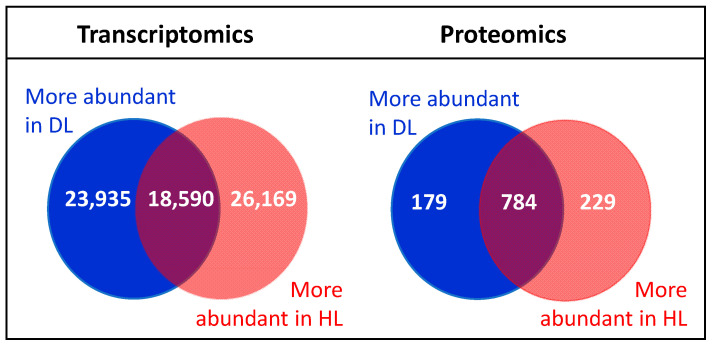
Identified differentially expressed genes (DEGs) and differentially abundant proteins (DAPs) between *R. serbica* HL and DL.

A total of 68,694 differentially expressed genes (DEGs) were obtained in *R. serbica* leaves upon desiccation (Supplementary Table S1). Applying a stricter threshold (*p*-value < 0.005 and abs (log2 FC) ≥ 2), we identified 23,935 and 26,169 genes that were upregulated and downregulated, respectively, in desiccated leaves (DL) compared with hydrated leaves (HL), while 18,590 genes were unaltered (Figure 1).

Using our *R. serbica* transcriptome data, differential TMT-based proteomic analysis allowed quantification of 1192 different protein groups which were identified after filtering with at least two unique peptides per protein (Supplementary Tables S2 and S3). Among them, 408 protein groups with a statistically significant difference (*p*-value < 0.05 and abs (FC) ≥ 1.3) in abundance between HL and DL were identified. In total, 229 protein groups were more abundant in HL and 179 in DL, while 784 were unaltered (Figure 1).

### 2.2. Functional Classification of DEGs and DAPs in R. serbica HL and DL 

GO-based classification of the biological processes indicated that a major proportion of DEGs between *R. serbica* DL and HL were related to transport, protein folding, N-cycle metabolism, carbohydrate, and lipid metabolism, followed by embryo development (involving genes encoding late embryogenesis abundant proteins, LEAPs), germin-like proteins (GLPs), reproduction, and cell proliferation (Supplementary Figure S1 and Table S4). The majority of the desiccation-affected proteins were associated with photosynthesis, proteostasis (namely protein synthesis), protein folding, stress response, and LEAPs, followed by carbohydrate metabolic processes, and transport (Supplementary Figure S1 and Table S5).

Among the various molecular functions, most of the DEGs were associated with oxidoreductase, transmembrane transporter, hydrolase, lyase, and DNA-binding transcriptional factor activities (Supplementary Table S4). According to the KEGG pathway database, most of the DEGs upon desiccation encoded proteins involved in signaling, starch and sucrose metabolism, and phenylpropanoid and flavonoid metabolism.

**Figure 2:**
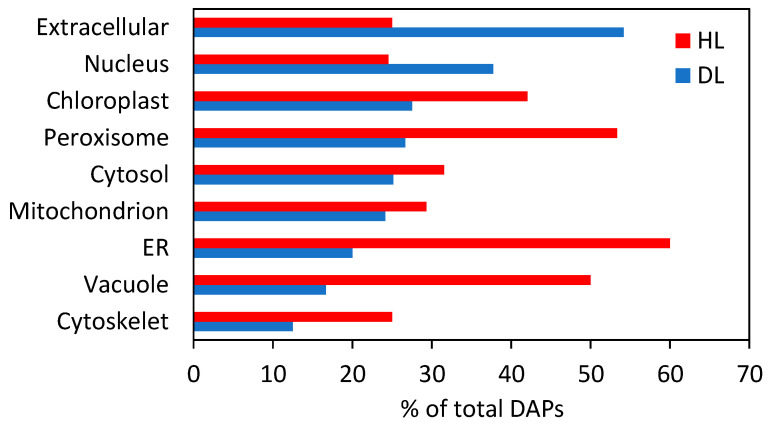
Predicted subcellular localization of DAPs in *R. serbica* HL and DL.

A large number of DEGs were markedly enriched in extracellular and cell wall regions and in thylakoids. Prediction of protein subcellular localization revealed that the identified DAPs were distributed within nine cellular compartments (Figure 2). The majority of DAPs were located inside the chloroplasts (42%), cytosol (32%), mitochondria (9%), and nucleus (8.3%) (Supplementary Table S2). Upon desiccation, the proportion of extracellular and nuclear proteins increased, while vacuolar, chloroplastic, and peroxisomal proteins, and those related to the endoplasmic reticulum and cytoskeleton, were reduced (Figure 2).

**Figure 3:**
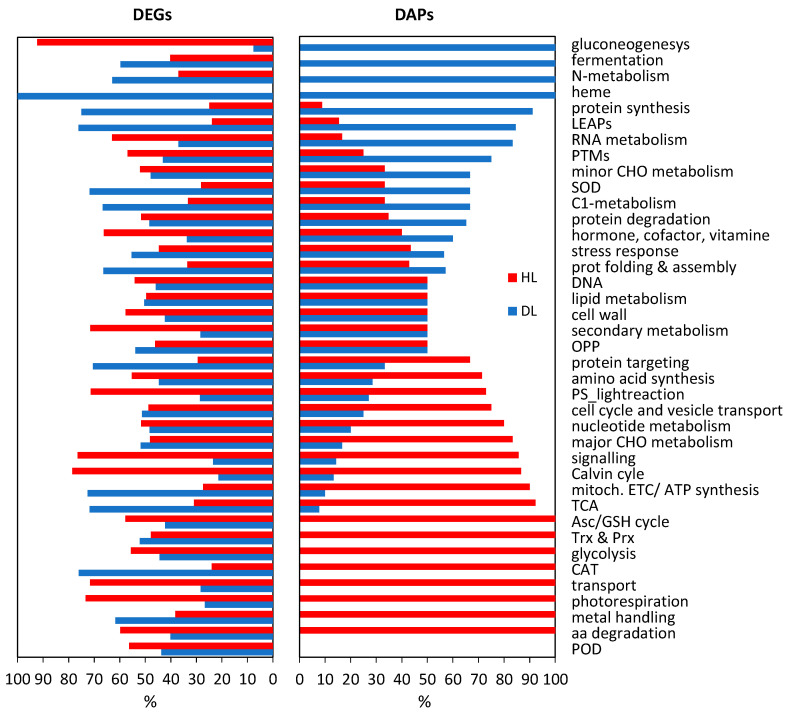
Functional classes of DEG and DAPs in R. serbica HL and DL. A detailed classification of DEGs and DAPs is given in Supplementary Tables S4 and S5.

Transcriptome and proteome data sets were classified into functional groups and compared to investigate whether changes in the abundance of transcripts and proteins correlated (Figure 3). It was notable that the majority of the DAPs and DEGs involved in photosynthesis (e.g., photorespiration, Calvin cycle, and light reactions), transport, secondary metabolism, and signaling, were less abundant in DL. However, proteins and transcripts associated with fermentation, N-metabolism, heme, protein synthesis, folding and assembly, C1-metabolism, as well as LEAPs, were more accumulated in DL (Figure 3).

### 2.3. Major Metabolic Pathways Affected in DL of R. serbica

**Figure 4:**
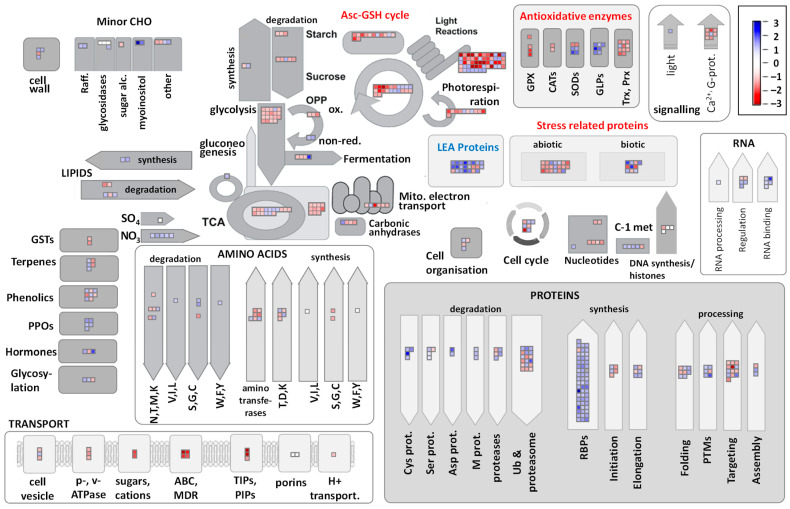
Mapman display of differentially abundant proteins (DAPs) in DL vs. HL, manually mapped according to TAIR mapping. Accumulated proteins in DL are indicated in blue, and accumulated proteins in HL are shown in red. AAs, amino acids; ABC, ATP-binding cassette transporters; Asc, ascorbate; CAT, catalase; CHO, carbohydrates; GLPs, germin-like proteins; GSH, reduced glutathione; GSTs, glutathione-S-transferases; MDR, multidrug resistance transporters; OPP, oxidative pentose-phosphate pathway; PIPs, plasma membrane intrinsic proteins; PPOs, polyphenol oxidases; Prx, peroxiredoxins; PTMs, post-translational modifications; ox-red, oxidoreductases; RBPs, ribosome-binding proteins; TCA, tricarboxylic acid cycle; TIPs, tonoplast intrinsic proteins; Trx, thioredoxins; Ub, ubiquitin; CHO, carbohydrates.

To obtain a better understanding of DAPs’ functional significance, we focused on those involved in well-characterized biochemical pathways of photosynthetic cells using Mapman, a bioinformatics tool commonly used for microarray data visualization [20]. Figure 4 shows that desiccation-inducible proteins were distributed among all metabolic pathways, but that some pathways had more DAPs than others. The major metabolic pathways affected by desiccation are discussed below.

#### 2.3.1. Photosynthesis

**Figure 5:**
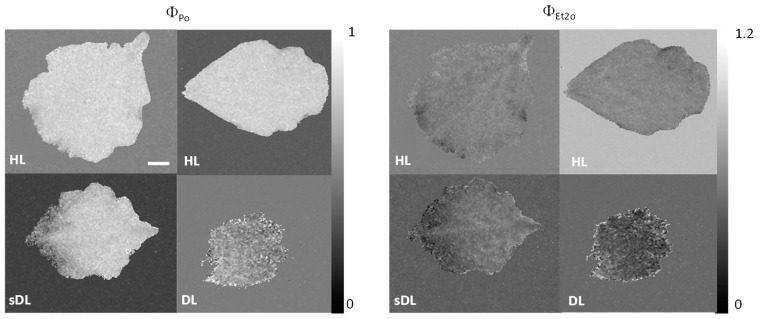
Representative images of the effects of dehydration on OJIP parameters in fully hydrated (HL; RWC~90%), semi-desiccated (sDL, RWC~60%), and desiccated (DL, RWC~15–20%) *R. serbica* leaves. Φ_Po_; equals Fv/Fm- maximum quantum yield of primary PSII photochemistry; Φ_Et2o_, a measure for the quantum yield of the electron transport flux from Q_A_ to Q_B_. The bar represents 1 cm.

**Figure 6:**
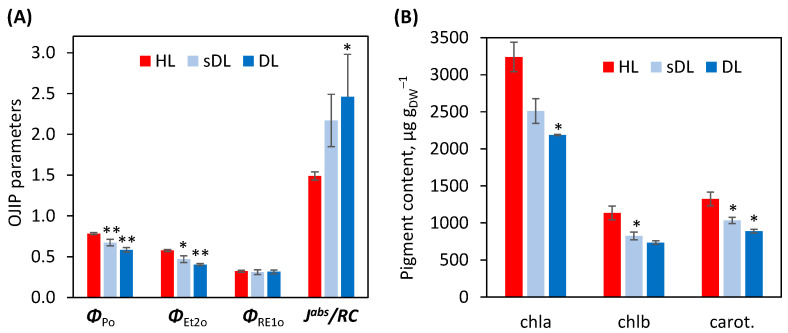
The influence of desiccation on OJIP parameters and the content of photosynthetic-related pigments in *R. serbica* leaves. (**A**) Φ_Po_; equals Fv/Fm- maximum quantum yield of primary PSII photochemistry; Φ_Et2o_, a measure for the quantum yield of the electron transport flux from Q_A_ to Q_B_, Φ_Re1o_ is the quantum yield of the electron transport flux until the PSI electron acceptors and J^abs^/RC is average absorbed photon flux per PSII reaction center. Asterisks denote significant differences between sDL and DL compared to HL according to the Mann–Whitney U-test (* *p* < 0.05, ** *p* < 0.01). (**B**) chla, chlorophyll a; chlb, chlorophyll b; carot, carotenoids. Asterisks denote significant differences between sDL and DL compared to HL according to the *t*-test (* *p* < 0.05). Values are shown as means ± SE.

Direct imaging of fast chlorophyll fluorescence kinetics (OJIP) showed a decreased maximum quantum yield of primary PSII photochemistry (Φ_Po_) in semi-desiccated leaves (sDL, ~60% relative water content, RWC) compared to fully hydrated ones, starting from the upper part of the leaf, while in DL (15–20% RWC) the decrease was homogenous, visible in the whole leaf (Figure 5). The higher sensitivity of mesophyll cells to desiccation than bundle sheath cells and the veins was visible in sDL regarding the quantum yield of the electron transport flux from Q_A_ to Q_B_ (Φ_ET2o_), while in DL both tissue types were affected to a similar extent. Inhibition of the efficiency of PSII, but not PSI, with desiccation, is visible also in Figure 6A, calculated for the whole leaf area. Desiccation induced an increase in average absorbed photon flux per PSII reaction center (J^abs^/RC) indicating a decrease in the number of active reactive centers (RCs), especially in DL, although this parameter was already 45% higher in sDL compared to HL.

Strong desiccation decreased the content of photosynthetic pigments, chlorophylls *a* and *b*, and carotenoids by 32–35% (Figure 6B). To monitor the dynamics of this change, sDL were analyzed, as well. Although the contents of all measured pigments tended to decrease in sDL compared with HL, the only statistically relevant change was noticed for chl *b* content (27% of reduction). No significant difference between sDL and DL regarding analyzed photosynthetic pigments content was observed.

**Figure 7:**
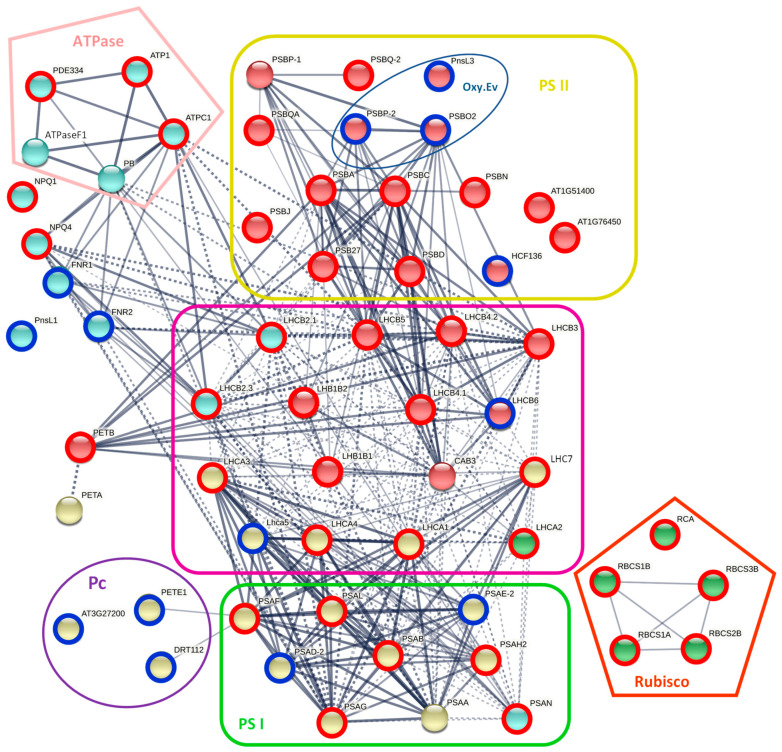
Graphical representation of protein-protein interaction network analysis of photosynthesis-related DAPs in *R. serbica* HL and DL performed by STRING software. K-means clustering into five clusters emphasizes distinct photosynthetic complexes. Connections between clusters are shown by dotted lines. A physical subnetwork is shown (connected proteins are part of the same physical complex). Line thickness indicates the strength of data support. For the minimum required interaction score, medium confidence was set at 0.400. Proteins rounded in blue were more abundant, while those rounded in red were less abundant upon desiccation. Abbreviations refer to symbols and annotation identifiers of the respective *A. thaliana* orthologous.

Consistent with the obtained results regarding pigments and photosynthetic efficiency, a 4.5 times lower number of photosynthesis-related proteins were detected among identified DAPs in DL (Supplementary Table S5). Specifically, 21 of 23 DAPs that mediate the Calvin cycle (such as Rubisco and its activase, phosphoglycerate kinase, and fructose-bisphosphate aldolases) and photorespiration (glycolate oxidase, glycerate dehydrogenase) were less abundant in DL compared with HL (Figure 7, Supplementary Table S5). Among 49 DAPs, constituents of the photosynthetic electron transport chain (PETC), 35 were less abundant upon desiccation. Two light-harvesting complex (LHC) subunits associated with the PSII (Rs_91513 and Rs_205281), two subunits of PSI (Rs_121438 and Rs_164136), a PSII stability/assembly factor (Rs_153379), three copper-ion-binding electron carriers (i.e., plastocyanins, Rs_90850, Rs_146509, and Rs_160542), and all four ferredoxin-NADP^+^-reductases (FNR, Rs_205228, Rs_156747, Rs_88126, and Rs_27514) were more abundant in HL compared with DL. Three proteins involved in oxygen-evolving at PSII were found to be more accumulated upon desiccation (Figure 7, Supplementary Table S5).

Regarding the transcriptomic results, 26 of 96, 18 of 61, and all 18 DEGs encoding components of PSI/II, light-harvesting complexes (LHC), and Rubisco, respectively, were upregulated upon desiccation (Supplementary Table S4). Interestingly, among 67 identified PETC-related DAPs, only 31 were represented by DEGs. Coherent changes within DAPs and DEGs were observed for LHC components, except for chlorophyll *a-b* binding protein 6a (Rs_20252), and PSI/PS II subunits, and except for the PSI reaction center (RC; Rs_121438) and PSI subunit IV (Rs_164136). Desiccation-induced changes of Rubisco, Rubisco activase, and ATPase-related transcripts and respective protein levels showed the same trends. On the other hand, transcript levels of three desiccation-accumulated oxygen-evolving enhancer proteins, plastocyanins, and FNRs were lower in DL, oppositely from protein levels (Supplementary Tables S4 and S5).

#### 2.3.2. Carbohydrate Metabolism

In order to evaluate the contribution of soluble sugars in desiccation tolerance in *R. serbica*, we measured the content of several of the most abundant hexoses, pentoses, disaccharides, oligosaccharides, and sugar alcohols. The total content of soluble sugars was tripled in DL upon desiccation (Table 1). The most abundant soluble sugars in *R. serbica* leaves were glucose, fructose, and sucrose, making ca. 28–32, 25, 14–16 mol % of the total sugars (Supplementary Table S6).

The highest statistically significant increase in DL compared with HL (more than four times) was obtained for galactose, rhamnose, stachyose, and arabinitol contents (Table 1). The glucose/fructose ratio slightly decreased upon desiccation. During desiccation, the molar percentage of glucose decreased ca. 5% (not statistically relevant), while mannitol increased from 0.3 to 3.9%; rhamnose and melibiose increased 40 times (from 0.1 to ~4%), gentiobiose from 0.02 to 6%, and stachyose from 0.02 to 3.5% (164 times) (Supplementary Table S6). Although stachyose synthase was not detected among DAPs, all eleven DEGs encoding this enzyme were more expressed in HL (Supplementary Table S4).

Four DAPs, annotated as galactitol-sucrose galactosyltransferase, involved in the synthesis of raffinose, tended to accumulate slightly more upon desiccation (20–30%) (Supplementary Table S5). Unlike HL, no DAP involved in sugar transport was found in DL.

Our transcriptomic data showed that some DEGs for key enzymes of starch biosynthesis were upregulated, and some were downregulated in DL. All three genes encoding starch synthase, as well as five out of twelve granule-bound starch synthases, and six of eleven 1,4-alpha-glucan-branching enzymes, were desiccation-responsive (Supplementary Table S4). Enzymes involved in starch degradation, such as α-amylase and α-glucan water dikinase, were mostly downregulated upon desiccation. Accordingly, the level of α-glucan water dikinase decreased in DL compared with HL (Supplementary Table S5). Considering β-amylase-related DEGs, eleven transcripts were upregulated in DL and five in HL.

A strong desiccation-dependent induction of several genes encoding proteins involved in sucrose degradation, such as sucrose synthases (Cluster-14775.53194; Cluster-14775.67439; Cluster-36760.0), fructokinases (Cluster-14775.41759—Rs_151532, Cluster-14775.46131—Rs_122669, Cluster-5400.0—Rs_22332, Cluster-14775.1477—Rs_168711, Cluster-14775.37835—Rs_120931, Cluster-14775.110729—Rs_168332), and one neutral and one cell wall invertase (Cluster-14775.100229—Rs_129413, Cluster-14775.61851—Rs_174460) were detected. Three sucrose synthases (Rs_93795, Rs_35712, Rs_35714), important for sucrose degradation in developing seeds, especially during the late maturation phase, were more abundant in HL than in DL, while one (Rs_35713) was more abundant in DL.

#### 2.3.3. Energy Production

*Glycolysis.* Genes that encode enzymes for the initial and committed steps of glycolysis, such as phosphofructokinase and phosphoglucomutase, were differentially expressed in HL and DL. Four out of the 11 DEGs encoding phosphoglucomutase were desiccation-inducible, as well as 11 out of the 12 DEGs encoding phosphofructokinase (Supplementary Table S4). All three DEGs for pyruvate kinases were upregulated in desiccated tissue. By contrast, two pyruvate kinases were less accumulated in DL, than in HL (Rs_171713 and Rs_205284; Supplementary Table S5). Desiccation upregulated only nine out of the 29 DEGs for phosphoenolpyruvate carboxylase (PEPC) involved in the conversion of PEP to oxaloacetate (OAA), a precursor of malate in crassulacean acid metabolism (CAM) photosynthesis. Similarly, three PEPCs were 40–60% less accumulated in DL than in HL (Supplementary Table S5).

*Fermentation*. The abundance of one aldehyde dehydrogenase (ALDH) involved in fermentation, as well as in detoxication and antioxidative protection, was doubled in DL (Supplementary Table S5). Furthermore, 21 out of 40 DEGs for alcohol dehydrogenase and 26 out of 38 DEGs encoding ALDH were significantly induced in DL (Supplementary Table S4).

*Oxidative and non-oxidative pentose-phosphate pathway*. The number of DEGs encoding the 6-phosphogluconolactonase, and 6-phosphogluconate dehydrogenase were higher in DL than in HL (seven of the eight, and six of the nine, respectively), while only eight out of the 21 glucose-6-phosphate-1-dehydrogenase transcripts were upregulated upon desiccation (Supplementary Table S4). Desiccation induced the accumulation of transketolase (Rs_169964) and its transcripts (Supplementary Table S5) involved in the non-oxidative part of the pentose-phosphate pathway. Specifically, half of the genes encoding different transketolases were desiccation-inducible (Supplementary Table S4).

*Tricarboxylic acid cycle(TCA)*. Most of the DEGs encoding the enzymes of the TCA cycle were induced by desiccation (72%). This was particularly emphasized in the case of fumarases (all), 2-oxoglutarate dehydrogenase complexes (all) and succinate dehydrogenases (16 of 21) (Supplementary Table S4). However, no desiccation-inducible enzymes involved in the TCA cycle were found (Supplementary Table S5). Only carbonic anhydrase (Rs_131324) accumulated more upon desiccation, while its transcript level slightly decreased. In addition, two mitochondrial NAD-malic enzymes (Rs_28856 and Rs_24043) were less abundant in DL compared with HL, although their transcript levels were unchanged (Supplementary Tables S4 and S5).

Regarding citrate synthase (CS), the first and rate-limiting enzyme in the TCA cycle, among seven DEGs encoding this enzyme, four were upregulated in DL (Supplementary Table S4), while only one isoform was found among DAPs, but its level slightly increased in HL (Supplementary Table S2). The slightly enhanced in vitro activity of endogenous CS was found already in sDL and it increased even more when RWC dropped below 50%, Table 2.

*Mitochondrial* ETC. Only three DAPs of the mitochondrial ETC (F1 subunits for two ATPases and uncoupling protein) were found in *R. serbica* leaves and all three were less abundant in DL (Figure 4). Most of the DEGs encoding proteins associated with complexes I-V were upregulated upon desiccation (Supplementary Table S4). Two genes encoding uncoupling protein (Cluster-14775.64964—Rs_127314 and Cluster-14775.67964—Rs_148751) were downregulated in DL. Almost an equal number of the up- and downregulated genes encoding ATP/ADP and other mitochondrial metabolite transporters were detected in DL and HL.

*Gluconeogenesis*/*Glyoxylate Cycle*. Genes mediating steps of this cycle, such as a pyruvate *ortho*-diphosphate dikinase, glyoxysomal citrate synthases, PEP carboxykinase, and Ala:glyoxylate aminotransferase were generally downregulated upon desiccation (Supplementary Table S4). Pertinent to this, among DAPs, only one identified PEP carboxykinase was slightly more abundant in HL (Supplementary Table S5).

#### 2.3.4. Antioxidative Metabolism

Strong alteration of genes and proteins involved in oxidative stress responses was observed in desiccated *R. serbica* leaves (Figure 4 and Figure S2; Supplementary Table S4).

*General stress response*. Genes encoding plant defense and stress-related proteins, including those associated with heat, dehydration, and salt stress, were differentially regulated (Supplementary Table S4). Namely, universal stress protein (Rs_79866), was more abundant in HL, while low-temperature-induced cysteine proteinase-like (Rs_120722) was more abundant in DL (Supplementary Table S2). Genes involved in proline biosynthesis were generally increased during dehydration, such as delta-1-pyrroline-5-carboxylate synthase (six of the eleven).

Interestingly, among the identified DEGs in HL and DL, 103 encoded various isoforms of class III peroxidases (PODs) (Supplementary Table S4). Among them, 58 were downregulated in DL. Unexpectedly, no POD was found among DAPs in HL and DL (Supplementary Table S5). Nineteen of the 25 DEGs for catalases (CATs) were more expressed in DL, while only two isoforms were observed among DAPs, both less abundant in DL. In addition, most DEGs encoding ascorbate oxidases were downregulated upon desiccation (Supplementary Table S4).

*Asc-GSH cycle*. Ascorbate (Asc) has a key role in hydrogen peroxide (H_2_O_2_) scavenging via the ascorbate-glutathione (Asc-GSH) cycle. The majority of detected DEGs encoding Asc peroxidase (APX), and 58% of the DEGs encoding other components of the Asc-GSH cycle, were lower upon desiccation (Supplementary Table S4). Within the identified DAPs, enzymatic components of the Asc-GSH cycle were either decreased or unaltered in DL compared with HL (Figure 4 and Figure S2). A similar number of DEGs encoding glutathione S-transferases (GST), peroxiredoxins, and thioredoxins were upregulated and downregulated in DL. Among DAPs, peroxiredoxins, thioredoxins, as well as thiol-based peroxidases, and two GSTs were less abundant in DL.

*Superoxide dismutase*. Six SOD isoforms were detected among DAPs in HL and DL. Interestingly, four Cu/ZnSOD isoforms were more abundant in DL, which correlated well with the respective DEGs. In contrast, MnSOD and FeSOD were more abundant in HL (Figure 4 and Figure S2), while DEGs related to FeSOD followed the same trend (Supplementary Table S4).

*Germin-like proteins*. Only two germin-like proteins (GLP)-encoding genes (Cluster-14775.5494—Rs_173035; Cluster-14775.5494—Rs_171415) were identified among DEGs and were upregulated in DL (Supplementary Table S4). However, all seven annotated GLPs within the DAPs were more accumulated in DL than in HL (Figure 4 and Figure S2). Multiple sequence and phylogenetic analyses showed high homology between these seven desiccation-induced GLPs (Supplementary Figure S3). Moreover, three homologues’ motifs were identified in these proteins, involving all three GLP-specific Boxes A-C.

*Secondary metabolism-phenolics*. The most abundant phenolic compound in *R. serbica* leaves was catechin, followed by epicatechin, and an unidentified compound, X337 (absorbance maximum at 337 nm) (Supplementary Table S7). Caffeic acid and its quinic ester, chlorogenic acid, were the most abundant hydroxycinnamic acids, while the levels of both flavon-3-ols, quercetin and kaempferol, were similar. Upon desiccation, the total content of identified phenolics was 40% higher in DL compared with HL, while the only statistically significant accumulation was obtained for compound X337.

Regarding DEGs encoding genes involved in shikimate, phenylpropanoid, and flavonoid biosynthesis pathways, almost all were downregulated in DL (Supplementary Table S4). Similarly, among identified DAPs, 2-hydroxyisoflavanone dehydratase (Rs_10942) was less abundant, while anthocyanidin-3-O-glucosyltransferase (Rs_14592) was more accumulated in DL than in HL (Supplementary Table S5).

Interestingly, five polyphenol oxidases (PPOs) tended to accumulate upon desiccation, and three of them were statistically more abundant in DL compared with HL (Figure 4, Supplementary Table S5). This observation was corroborated by the increased activity of PPOs extracted from *R. serbica* leaves during desiccation (Table 2). The maximal in vitro PPO activity was reached in DL. However, DEGs encoding these enzymes were more expressed in HL (11 out of the 12, Supplementary Table S4).

#### 2.3.5. Late Embryogenesis Abundant Proteins

Very recently, we have listed 359 LEAPs in *R. serbica*, 318 containing more than 100 amino acids [21]. In this study, we identified 88 DEGs encoding LEAPs. Among the upregulated LEAP-encoding genes, almost 21% encoded proteins that belonged to the LEA4.3 protein family subgroup and almost 14% belonged to the LEA1 protein family group, while most of the desiccation-downregulated genes belonged to the LEA2 gene family group.

Quantitative TMT-based proteomic analysis confirmed this trend (Table 3). In total 26 LEAPs were annotated, while 22 were desiccation-inducible and predicted to be distributed mostly in the nucleus, chloroplasts, and mitochondria. Eighteen of them belonged to LEA4, two to dehydrin, and one to the LEA1 protein family group. Three LEA2 and one seed maturation protein (SMP) family members were less abundant in DL than in HL (Table 3).

#### 2.3.6. Cell Wall Remodeling

Three pectin methylesterases (PMEs) were 10–30% more abundant in DL compared with HL (Supplementary Table S5). These enzymes modify cell walls via pectin demethylesterification. In total, 27 and 45 DEGs annotated as PMEs and PME inhibitors (PI) were obtained in HL and DL (Supplementary Table S4). Thirteen PMEs, and only three PIs DEGs encoding PMEs, were upregulated upon desiccation. Genes encoding pectin/pectate lyase (PL) and polygalacturonases (PGs) were specifically regulated in both HL and DL.

All seven transcripts encoding cellulose synthase (within DEGs), as well as one protein product, were less abundant in DL than in HL (Supplementary Tables S4 and S5). Eleven of the twelve DEGs encoding expansins were downregulated in DL (4–8 times). All seven DEGs encoding leucine-rich repeat extensin-like proteins, one extensin, eight leucine-rich repeat proteins, and six hydroxyproline-rich glycoproteins were downregulated in DL (Supplementary Table S4). Among cell-wall-related genes, the lowest expression levels (8–10 times) were exhibited by those encoding three repetitive proline-rich cell wall proteins, detected in DL. On the other hand, two glycine-rich proteins, (Rs_28695 and Rs_140483) were more abundant upon desiccation (Supplementary Table S2).

**Figure 8:**
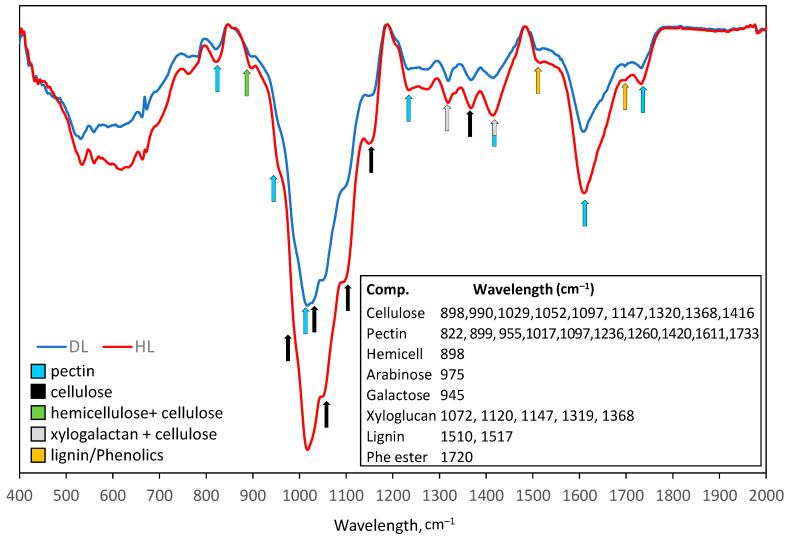
FTIR spectra of the cell walls of *R. serbica* HL (red line) and DL (blue line). The average spectra of seven HL and seven DL samples (biological replicates) are shown. Peaks corresponding to cellulose, pectin, hemicellulose, xylogalactan, and lignin (phenolic ring and phenolic esters) are pointed.

Fourier transform infrared (FT-IR) spectroscopy was used to profile plant cell walls. Overlaid DL and HL cell wall FTIR spectra are shown in Figure 8. The bands related to cellulose, such as a symmetric CH_2_ vibration at 1416 cm^−1^, CH_2_ bending vibration at 1368 cm^−1^, CH_2_ wagging vibration at 1320 cm^−1^, O–C–O asymmetric stretching glycosidic link vibration at 1147 cm^−1^, C–OH stretching secondary alcohol vibration at 1052 cm^−1^, C–OH stretching primary alcohol at 1029 cm^−1^, and C–C stretching (C6-H2-O6) at 990 cm^−1^ were found in both cell wall samples (references are given in [22]). The bands at 1733 cm^−1^ (C=O stretching vibration of alkyl ester), 1611 cm^−1^ (COO^–^ antisymmetric stretching vibration of polygalacturonic acid), 1420 cm^−1^ (COO^–^ stretching), 1260 and 1236 cm^−1^ (C–O stretching), 1097 cm^−1^ (C–O and C–C stretching), 1017 cm^−1^ (C–O, C–C stretching, e.g., C_2_–C_3_, C_2_–O_2_, C_1_–O_1_), 955 cm^−1^ (CO bending), 822 cm^−1^ (ring vibration), are characteristic for pectin. The peaks at 1510 and 1517 cm^−1^ are characteristic for lignin and those at 1720 cm^−1^ for phenolic esters (Figure 8). The bands at 1072 cm^−1^ (C–O stretching, C–C stretching), 1120 cm^−1^, 1147 cm^−1^, 1319 cm^−1^, and 1368 cm^−1^, are typical for xyloglucans [23]. The peak at 898 cm^−1^ is related to cellulose, hemicellulose, and pectin.

Briefly, peaks indicating cellulose, hemicellulose (overlapped with cellulose), pectin, xyloglucans, and lignin were higher in HL than in DL (Figure 8).

## 3. Discussion

This is the first comprehensive proteomic and transcriptomic study of hydrated (HL) and desiccated leaves (DL) of the resurrection plant *R. serbica.* Results from our study showed that desiccation induced global changes in the transcriptome and proteome of *R. serbica* leaves, implying a unique strategy to cope with the cellular anhydrous status.

### 3.1. Comparison of Proteomic and Transcriptomic Data 

Based on the proportion of DEGs/DAPs involved in various metabolic pathways (Figure S1), it can be concluded that the most abundant proteins, such as ribosomal, photosynthesis- and stress-related, as well as late embryogenesis abundant proteins (LEAPs), were mostly detected by proteomic analysis, compared with those, generally less abundant, linked to signaling and transport processes. This can be explained by the limits in the sensitivity of current protein and RNAseq analysis technology, as suggested by Ross et al. [24].

Moreover, our proteomic and transcriptomic results revealed discrepancies, such as in the case of mitochondrial electron transport (MET) and ATP production, gluconeogenesis, glycolysis, tricarboxylic acid cycle (TCA), and enzymatic H_2_O_2_ scavengers, catalases (CAT) and peroxidases (PODs) (Figure 3). A weak correlation between the abundance of transcripts and their corresponding proteins has been observed, particularly during developmental changes [24] and under stress [25]. The reasons for this lie in the post-transcriptional regulation, mRNA half-lives, protein turnover, and posttranslational modifications (PTMs) [24,26,27]. The discrepancy between the proteomic and transcriptomic results was also reported for photosynthetic genes and proteins that are encoded by nuclear and chloroplastic genomes [27]. Similarly, a general low coherence between transcript and protein abundance was recently observed in the case of the resurrection plant *C. plantagineum* [28]. Therefore, we propose that the poor correlation between transcript and protein levels during desiccation in *R. serbica* can be attributed to dynamic posttranscriptional and PTMs, as well as to different mRNA half-life and protein turnover (see later), enabling temporal and compartment-specific regulation of specific processes crucial for desiccation tolerance.

### 3.2. Cell Wall Remodeling

Morphological adaptations of *R. serbica* leaves during dehydration (10–15 days) include folding inward so that the hairy abaxial leaf side becomes exposed to the sunlight. In this way, the possibility of reactive oxygen species (ROS) generation at the photosynthetic electron transfer (PET) chain is minimized [13,29]. During desiccation and rehydration, the cell wall of resurrection plants extensively shrinks and folds upon desiccation to avoid damage caused by mechanical stress [6]. Such cell wall remodeling, specific for the resurrection species, involves enzymes responsible for the composition of the cell wall polysaccharides (e.g., pectins, arabinogalactans, xyloglucans) and proteins (e.g., expansins, arabinan-, proline- and glycine-rich proteins) [10,30].

Pectin, cellulose, hemicellulose, and xyloglucans were identified as polysaccharide components of the *R. serbica* cell wall and they decreased upon desiccation (Figure 8). The reduction in cellulose can be correlated with the lower abundance of cellulose synthase upon desiccation (Supplementary Table S5, Figure 4). On the other hand, the abundance of three pectin methylesterase (PMEs) increased upon desiccation (Supplementary Table S5). PMEs demethylate carboxyl functional groups in galacturonic acids of pectin polymers. Similarly, as in *R. serbica*, 33 and 48 transcripts were annotated as PMEs and PIs, respectively, and only one PME transcript was upregulated upon desiccation in the resurrection plant *C. plantagineum* (no PIs were desiccation-inducible) [31]. An increased level of demethylated pectins has also been shown in some resurrection species during desiccation [32]. While an increase in blockwise demethylation results in Ca^2+^-dependent cell wall stiffening, random demethylation assisted with pectin lyase and polygalacturonase activities leads to cell wall loosening [33].

Some resurrection species accumulate arabinose-rich polymers (pectin-arabinans, arabinogalactan proteins, and arabinoxylans), while some, such as Craterostigma spp., adjust cell wall plasticity via altering xyloglucan content and pectin methylation degree to enable cell wall flexibility during desiccation [30,34]. Besides PMEs and PIs, in *A. thaliana*, the degree of pectin methylesterification is controlled by the action of subtilisin-type Ser proteases [35]. Indeed, a single extracellular subtilisin-like protease (Rs_39172, Supplementary Table S2) was 50% more abundant in DL. Moreover, polysaccharides and transcripts encoding cell wall proteins (e.g., expansins, extensins, also known as proline- and hydroxyproline-rich glycoproteins) were downregulated (some completely suppressed) in DL of *R. serbica*. Extensins are generally involved in cell wall reinforcement (i.e., in the crosslinking of cell wall polymers), while expansins participate in pH-dependent cell wall loosening. Similarly, as in *R. serbica*, glycine-rich proteins, CpGRP1 and BhGRP1 accumulate in desiccated leaves of resurrection plants *C. plantagineum* and *B. hygrometrica*, respectively [36,37]. The latter was proposed to be correlated with cell wall flexibility [36].

Taken together, the reduced content of all cell wall polysaccharides and lignin, as well as the proposed decreased methylation in *R. serbica* DL, do not provide sufficient information to conclude whether the cell wall is stiffening or loosening during desiccation. Therefore, more detailed analysis, particularly measuring the activity of cell wall modifying enzymes and ROS generation in cell wall microdomains, needs to be undertaken to reveal the complex and multi-level regulated DL folding process.

### 3.3. Germin-like Proteins

One of the most striking results found upon desiccation in *R. serbica* leaves was the increased abundance of seven germin-like proteins (GLPs). Germins and GLPs are multifunctional plant apoplastic glycoproteins that might exhibit oxalate oxidase (OXO), Mn superoxide dismutase (MnSOD), ADP glucose pyrophosphatase/phosphodiesterase, and polyphenol oxidase (PPO) activities, as well as act as rhicadhesin receptors [38]. Germin-like protein GLP5A was found to accumulate in dried leaves of the resurrection plant *B. hygrometrica* [36]. Desiccation-inducible CpGLP1 cell wall protein weakly interacts with pectins and exhibits SOD activity, suggesting that it may be involved in H_2_O_2_/O_2_^•–^ regulation in another resurrection plant *C. plantagineum* [31]. Multiple sequence and phylogenetic analysis showed 28–39% of identity and 48–61% of homology of CpGLP1 and seven desiccation-induced RsGLPs (Supplementary Figure S3). Thus, we proposed that GLPs in *R. serbica* DL might exhibit SOD-activity and be the important producers of H_2_O_2_ in the cell wall.

H_2_O_2_ might be the fine regulator of cell wall response to desiccation in several ways. Cell wall peroxidases (PODs) use H_2_O_2_ as a co-substrate to mediate crosslinking of cell wall components, but they can also produce hydroxyl radicals (HO^•^) from H_2_O_2_ resulting in cell wall loosening [39]. In addition, HO^•^ can be produced non-enzymatically from H_2_O_2_, e.g., via the Haber–Weiss reaction in the presence of the metals bound to pectins and xylans [40]. We have previously shown transiently increased activity of specific PODs in semi-DL (RWC 50%) [5]. However, although 45 of 103 POD-related genes were upregulated in DL (Supplementary Table S4), no protein product was observed among DAPs (Figure 3). Keeping in mind the lignin decrease during desiccation, H_2_O_2_ could primarily serve as a source of HO^•^, which, in turn, may cause cell wall loosening in a POD-independent way [41]. Finally, H_2_O_2_ is an important messenger in salicylic acid and/or jasmonic acid signaling and this role in desiccation tolerance needs to be considered as well.

### 3.4. Photosynthesis

As a typical homoiochlorophyllous desiccation-tolerant plant, *R. serbica* retained around 63% of chlorophyll *a*, as well as chlorophyll *b* and carotenoid contents, thus keeping a constant chl*a*/*b* ratio during desiccation (Figure 6) [17]. This strategy allows rapid recovery upon rewatering, but it also increases the probability of ROS accumulation at PET. Our transcriptomic and proteomic analysis showed a strong decrease in protein subunits of the light-harvesting complexes (LHC), PSI, PS II, and Rubisco. This is in accordance with transcriptomic and proteomic data obtained for poikilochlorophyllous desiccated *C. plantagineum* leaves [28]. Similarly, in the poikilochlorophyllous monocotyledonous resurrection plant *Xerophyta humilis*, PSII reaction center (RC) proteins were downregulated in response to dehydration [42].

Interestingly, three of four oxygen-evolving enhancer (OEE) proteins were more abundant in DL than in HL (Figure 7). The OEE2 that stabilizes the Mn4Ox cluster at PSII, and two other peptides (33 and 17 kDa) constitute the oxygen-evolving complex (OEC) [43]. Increased accumulation of the OEC precursor, has also been reported during desiccation and rehydration in the phenotypically similar resurrection plant *B. hygrometrica* [44].

Given the limitation of CO_2_ fixation due to closed stomata and subsequent PET overreduction [28], and increased leakage of photosynthetic electrons to O_2_ in the Mehler reaction at PSI during desiccation [3], it is possible that phosphorylated OEE may modulate H_2_O_2_/O_2_^●–^ formation [43]. Moreover, the OEC structure stabilization by OEEs during dehydration might be an important prerequisite for rapid function regain upon rewatering.

Photosynthesis and respiration are particularly susceptible to oxidative stress during dehydration [45]. At the beginning of *R. serbica* dehydration, the photochemical efficiency of PSII (Φ_Po_) slightly decreased (Figure 6), as previously obtained for *R. serbica* and *H. rhodopensis* [29]. However, in *B. hygrometrica*, Φ_Po_ was not significantly affected at the beginning of the desiccation [44].

Our OJIP analyses showed that *R. serbica* desiccation induced a decrease in RC number and PSII efficiency reduction, without affecting P700 (PSI). This is in accordance with PSII activity inhibition observed upon desiccation in *B. hygrometrica* [44] and with chlorophyll fluorescence kinetics in *H. rhodopensis* [14]. The desiccation-induced accumulation of four FAD/NAD(P)-binding oxidoreductase (FNR) isoforms (Supplementary Table S4) supports previous findings. FNR is involved in the cyclic electron transport (CET) around PSI, protecting both photosystems from stromal over-reduction by increasing the ATP/NADPH ratio [46]. Taken together, dehydration-induced CET around PSI is likely to protect PSII against excessive excitation pressure and maintain photosynthetic ATP synthesis. Desiccation-triggered CET has also been suggested for other resurrection plants, such as *Paraboea rufescens* [47], *B. hygrometrica* [48], *H. rhodopensis* [49], and *C. plantagineum* [28].

### 3.5. Energy Metabolism

An overall decrease in the accumulation of transcripts and/or proteins associated with the glycolytic process in *R. serbica* DL was the opposite of the results obtained in *C. plantagineum* [28]. A decreased abundance of three phosphoenolpyruvate carboxylases (PEPC) upon desiccation in *R. serbica* suggested weaker carbon flux from the breakdown of glucose/fructose into the tricarboxylic acid (TCA) cycle. Although in *C. plantagineum*, PEPC accumulated more than five-fold, the desiccation-induced TCA cycle in *R. serbica* was in accordance with data obtained for *C. plantagineum* [28]. In addition, six NADP-dependent malic enzymes (NADP-MEs) and two NAD-MEs were less accumulated upon desiccation in *R. serbica*, contrasting with the several times upregulated gene expression of six NADP-dependent malic enzymes during early dehydration in *C. plantagineum* [28]. Therefore, we could not confirm desiccation-induced shifting to crassulacean acid metabolism (CAM)-type photosynthesis in *R. serbica*, as demonstrated previously in the case of *C. plantagineum* and *H. rhodopensis* [50]. However, this conclusion should be reconsidered in future research, since the transcript abundances are not always coherent with changes in protein levels, as we discussed above [27]. It is still possible that the glycolytic and respiratory enzymes of *R. serbica* are more sensitive to dehydration and have a fast turnover that could be compensated for by the accumulation of the respective transcripts.

Interestingly, desiccation doubled the abundance of an aldehyde dehydrogenase ALDH3 (Supplementary Table S5). Similarly, Kirch et al. [51] reported the drought-inducible plastid ALDH3 in *C. plantagineum* belonging to a new, different plant ALDH class. ALDH3 was suggested to possess a detoxification function, as it may oxidize medium-size aldehyde substrates generated by the peroxidation of plastid lipids.

### 3.6. Soluble Sugars and Proline

Adaptation to cellular water loss implies the increased biosynthesis of osmoprotectants, such as polyols, proline, sucrose, raffinose, trehalose and other sugars that accumulate in many tissues [8,12]. Pertinent to this, most of the measured sugars increased in abundance in DL of *R. serbica* compared with HL (Table 1). Sucrose was the major soluble sugar in *R. serbica* leaves and it accumulated during dehydration, as reported by Živkovic et al. [52]. Sucrose has a crucial role in the glassy state in osmotic protection to survive desiccation [4,53]. Similarly, as in *R. serbica*, the levels of mannitol, maltose, galactitol, and trehalose increased in desiccated leaves of *C. plantagineum* [28]. Besides being osmoprotectants, oligosaccharides can have signaling and antioxidative functions. Trehalose and trehalose-6-phosphate act as signaling molecules that promote starch degradation and biosynthesis, respectively [54]. In addition, the content of raffinose, and several sugar alcohols with HO^•^ scavenging potential [55], significantly increased during desiccation in *R. serbica*.

A slight increase in the glucose/fructose ratio in DL may indicate more intensive degradation of starch compared with sucrose degradation. This is correlated with the increased levels of maltose and enzymes involved in starch catabolism (Supplementary Table S5).

The increase in proline, an important osmoprotectant in DL of *R. serbica* [39], correlated well with the desiccation-inducible upregulation of genes involved in proline biosynthesis (Supplementary Table S4).

### 3.7. Chloroplastic Antioxidants and Phenolics

Specific components of the antioxidative system were stimulated in *R. serbica* DL. In contrast to our results related to ascorbate peroxidase (APX) abundance (Figure 4) and activity [7], increased APX activity during dehydration has been reported in *C. wilmsii* and *X. viscosa* [53]. All three SOD types, Cu/Zn-, Mn-, and FeSOD, were identified both by transcriptome and proteome analyses. Among them only four chloroplastic Cu/ZnSOD isoforms were more abundant in DL compared with HL. The major H_2_O_2_ scavenging system in chloroplasts is the ascorbate-glutathione (Asc-GSH) cycle. Moreover, the role of 2-Cys peroxiredoxins (PRXs) as major components in the water-water cycle was proposed as an alternative to thylakoid-bound APX [56]. Keeping in mind the lower abundance of the Asc-GSH cycle enzymatic components, as well as thioredoxins, peroxiredoxins, and glutathione peroxidases (Figure 4), the role of Cu/ZnSOD becomes particularly important for O_2_^•–^ dismutation to H_2_O_2_ in chloroplasts. It has been suggested that increased H_2_O_2_ accumulation during the later phases of dehydration might serve as a signal for the increase in Asc and GSH pools in *R. serbica* leaves [57].

*R. serbica* leaves are exceptionally rich in phenolic compounds, requiring special protocols for proteins and RNA extraction [58]. The accumulation of polyphenols in *R. serbica* leaves has already been observed during desiccation, oxidative stress upon rehydration, and UV-B exposure [59,60,61]. Together with Asc, ~40% increased phenolics induced by desiccation (Supplementary Table S6) can participate in H_2_O_2_ scavenging by class III peroxidases (PODs) in the vacuoles and apoplast [62].

Notably accumulated polyphenol oxidases (PPOs) correlated with several-fold higher PPO activity and induction of new PPO isoforms [60], as well as two additional quinone-oxidoreductases, might be involved in the relaxation of the overreduced PET during desiccation in *R. serbica* leaves. Moreover, the increase in the PPO protein levels and enzymatic activity was reported as one of the first responses to desiccation in the leaves of the resurrection plant *B. hygrometrica* [44]. PPO is found in the lumen of thylakoids, physically separated from vacuoles, where the majority of its endogenous substrates (caffeic and chlorogenic acid in the case of *R. serbica* PPO [60]) are located [63]. Its close association with photosystems contributes to the hypothesis that PPO can function as an oxygen buffer, by spending O_2_ to generate *o*-diphenols from monophenols and to oxidize *o*-diphenols to *o*-quinones [63]. In addition, PPO coupled with Mehler peroxidase activity uses the electrons from *o*-diphenols to reduce dehydroascorbate to Asc. This mechanism coupled with the Asc-GSH cycle can favor NADPH oxidation and relax the overreduced plastoquinol pool.

### 3.8. Late Embryogenesis Abundant Proteins

The induction of late embryogenesis abundant proteins (LEAPs) is considered an essential part of the vegetative desiccation tolerance strategy in resurrection plants [13,15]. Studies conducted on several recombinantly produced LEAPs from different species, including Arabidopsis, suggest that LEAPs can be involved in water binding, ion sequestration, and the stabilization of membranes and enzymes during freezing or drying [64,65].

The majority of *R. serbica* desiccation-induced LEA genes and proteins belong to the LEA4, LEA1, and dehydrin family groups (Table 3, [21]). All three LEA protein family groups are very hydrophilic and display an unusual preponderance of glycine, lysine, and glutamate residues, similar to the same LEA protein families in other species [19]. On the other hand, LEAPs that were less abundant in DL than in HL belong to the atypical LEA2.2 protein group characterized by a higher portion of hydrophobic amino acids, a compact β-barrel domain, and a single amphipathic α-helix [21].

We have recently shown that the *R. serbica* LEA4 protein family group has a high propensity to form so-called A-type α-helices, containing positive, negative, and hydrophobic faces [21]. This feature enables them to immerse laterally within the membrane, reinforcing the membrane in the dry state [65]. Thus, mitochondrial LEA4, Rs_52085 (87% of the sequence length tends to adopt α-helix, A-type) can stabilize the inner membranes of these important organelles. However, the lipid composition of the inner envelope membrane of chloroplasts and thylakoids comprises a high proportion of neutral galactolipids, and only 8–10% phospholipids [66]. Therefore, desiccation-inducible chloroplastic LEA4 proteins (e.g., Rs_3451) are more likely to interact with desiccation-sensitive proteins in chloroplasts, particularly with PET components. In the case of nuclear LEA4 proteins, such as upregulated Rs_136891, the gathering of positive residues to form a negatively charged strip of the A type of α-helix can be important for binding and stabilizing DNA.

Desiccation-induced accumulated lysine-rich *R. serbica* dehydrins can bind to the anionic phospholipid vesicles [67]. In addition, similarly to Arabidopsis, *R. serbica* dehydrins contain a high proportion of histidine residues, involved in Zn^2+^ chelation required for binding to DNA [68]. Finally, LEA1 proteins from *R. serbica* exhibited a high propensity to form amphipathic α-helices at the N-terminus and random coil at C-terminus [21]. Two members of the LEA1 family group from the resurrection plant *B. hygrometrica* overexpressed two LEA1 desiccation-inducible proteins contributing to better tolerance to drought in tomato plants, as evidenced by more preserved membranes and proteins associated with photosynthesis and ROS scavenging [69].

Due to the disordered nature and structural plasticity of the desiccation-induced dehydrins, LEA4, and LEA1 proteins in *R. serbica*, they can act as ‘molecular shields’ and affect protein aggregation under water deficit and/or macromolecular crowding [70,71]. Secondly, as intrinsically disordered proteins, these LEAPs can endorse desiccation tolerance by induction of the liquid-liquid phase separation and formation of non-membranous organelles, i.e., intracellular proteinaceous condensates [16,72].

### 3.9. Proteostasis

Protein content is dependent on its turnover and the balance between synthesis and degradation in lysosomes and proteasomes [24]. Our proteomic data undoubtedly showed increased protein translation upon desiccation. This is in accordance with our previous report of de novo protein synthesis in *R. serbica* (e.g., several polypeptides ~15, ~25, ~35, ~60 kDa) [5]. The early appearance of new proteins is consistent with a study of protein synthesis in the desiccation-tolerant *B. hygrometrica* [44]. In addition, dynamic changes in protein phosphorylation were induced in the resurrection plants during desiccation [73].

The majority of generally abundant proteins, such as those related to photosynthesis, cell wall and energy production, were less abundant in DL. However, 24 LEAPs, six GLPs, three PPOs, and four Cu/ZnSODs were significantly accumulated in DL. Therefore, we suggest that highly abundant photosynthetic proteins, as well as expansins and extensins rich in proline, glycine, and polar amino acids, are being recycled through desiccation responsive proteasomes to allow rapid de novo synthesis of LEAPs by chaperones and ribosome binding proteins accumulated in DL. In addition, pre-existing proteins can be post-translationally modified, as indicated by the increased levels of the proteins involved in PTM (Figure 3). Desiccation-induced proteases, such as the already mentioned subtilisin-like protease, might be involved in the regulation/activation of target proteins (e.g., the abovementioned PPOs, [74]) rather than in protein degradation. We speculate that upregulated genes and accumulated proteins under desiccation are protected against aggregation by strongly accumulated LEAPs. Substantial enzyme conservation is beneficial for the rapid resumption of metabolic activity in resurrection plants [75].

## 4. Materials and Methods

### 4.1. Plant Material and Experimental Conditions

The resurrection plants *Ramonda serbica* Panč. were collected from their natural habitat in a gorge near the city of Niš in south-eastern Serbia. Desiccation was induced as described in Veljović-Jovanović et al. [5]. Relative water content (RWC) was measured at regular intervals during dehydration, according to the formula: 100 × [(hydrated weight—dry weight)/(saturated hydrated weight–dry weight)]. Fully expanded leaves from the middle part of the rosette, comparable in size (4–5 per plant) of five hydrated plants (with approximately 90% of relative water content, RWC) and of five desiccated plants (15% RWC) were harvested, frozen in the liquid nitrogen and stored at −80 °C until analysis.

### 4.2. RNA Isolation, cDNA Library Construction, Sequencing, and De Novo Transcriptome Assembly

The procedures for total RNA extraction and purification from hydrated (HL) and desiccated (DL) leaves of *R. serbica*, as well as for de novo transcriptome assembly were recently described in detail [21].

### 4.3. Functional Annotation and Gene Expression Analysis 

A comprehensive transcriptomic analysis of *R. serbica* HL and DL gene expression upon desiccation has been recently performed by our team and a link for the complete *R. serbica* de novo transcriptome database is reported in Pantelić et al. [21]. Raw data from this article can be found in the Short Read Archive database at NCBI under accession numbers SRR18015613 and SRR18015612 (Bioproject accession no. PRJNA806723 and Sample accession no. SAMN25859880). An overview of data production quality, length distribution, number of transcripts and unigenes, and annotated unigenes are presented in Pantelić et al. [21].

Functional annotation of the unique assembled transcripts was performed using the following databases: NCBI non-redundant (NR) protein sequences, NCBI NR nucleotide sequences, Protein family (Pfam) database, Clusters of Orthologous Groups of proteins (KOG/COG), Swiss-Prot, Kyoto Encyclopedia of Genes and Genome (KEGG) Ortholog database, and Gene Ontology, and GO by GOseq R package, as described in Pantelić et al. [21]. The read counts were adjusted by the edgeR algorithm through one scaling normalized factor for each sequenced library. To identify differentially expressed genes (DEGs) between *R. serbica* HL and DL, the expression level of each transcript was calculated according to the FPKM method. Estimation of gene expression levels was carried out by RSEM for each sample. Clean data were mapped back onto the assembled transcriptome, while the read count for each gene was obtained from the mapping results. DEG analysis between HL and DL was performed using the DEGseq R package. The *p*-values were adjusted using the Benjamini and Hochberg methods. A corrected *p*-value of 0.005 and |log2^(Fold Change)^| of 2 were set as the threshold for significantly differential expression [21].

Functional enrichment analysis including GO and KEGG terms compared to the whole-transcriptome background was performed. GO enrichment analysis of differentially expressed genes was implemented by the GOseq R package in which gene length bias was corrected. GO terms with corrected *p*-value < 0.05 were considered significantly enriched by differentially expressed genes [21]. Statistical enrichment of differentially expressed genes in KEGG pathways was tested using KOBAS software.

### 4.4. Protein Extraction, TMT Labeling and SCX Fractionation

Proteins from three HL and DL pooled replicates (a mix of leaves from three plants used for transcriptomic analysis) were harvested, immediately transferred to liquid nitrogen, and extracted using the previously optimized protocol for *R. serbica* [75]. Protein concentration was determined by Bradford’s assay [76]. For each sample, 50 µg of proteins was digested through the filter-aided sample preparation method as described in Vidović et al. [75]. Obtained peptides were dissolved in 0.1% formic acid (FA) prior to desalting with C18 cartridges (Sep-Pack, C18, Waters, Milford, MA, USA) and dried by the Speed-Vac system.

Purified peptides were labeled with 6-plex TMT reagents (Thermo Fisher Scientific, MA, USA; tags 126, 127, and 128 for HL and 129, 130, and 131 for DL) according to the manufacturer’s protocol. Briefly, 8 μL of each TMT label (0.8 mg in 40 μL of ACN) was added to each peptide extract [77]. The reaction mixture was incubated at room temperature for 1 h and quenched by the addition of 8 μL of 5% (*w*/*v*) NH_2_OH for 15 min. To verify the labeling efficiency, each sample was purified with zip-tip C18 tips and analyzed by liquid chromatography-tandem mass spectrometry (LC-MS/MS) as described below. Acquired data were searched with the Mascot search engine setting TMT labeling as variable modification. No unlabeled peptides were identified from the search and all the peptides were correctly modified at the N-terminus and each lysine residue. Finally, all six labeled protein solutions were mixed in equal amounts and dried by the Speed-Vac system.

To reduce the complexity of the peptide mixture and increase the MS detection probability, labeled peptides were fractionated by strong cation exchange (SCX) fractionation using an SCX cartridge (Applied Biosystems, Foster City, CA, USA) and following the manufacturer’s protocol. Pooled labeled proteins were resuspended in 500 µL of a buffer consisting of 5 mM KH_2_PO_4_, 25% ACN, pH 2.9, loaded into an SCX cartridge, and eluted in six steps by increasing KCl concentrations (50, 100, 150, 200, 250, and 350 mM KCl) at 50 μL min^–1^ flow rate [77]. Each SCX fraction was vacuum dried, suspended in 0.1% FA, and desalted using C18 cartridges. Samples were vacuum dried and resuspended in 0.1% FA to an estimated concentration of 1 mg mL^–1^ immediately prior to liquid chromatography coupled to tandem mass spectrometry (LC-MS/MS) analysis.

### 4.5. Liquid Chromatography Coupled to Tandem Mass Spectrometry 

The obtained fractions were analyzed with an LTQ-Orbitrap XL mass spectrometer (Thermo Fisher Scientific, Bremen, Germany) coupled online with an Ultimate 3000 nano-HPLC (Dionex-Thermo Fisher Scientific, Sunnyvale, CA, USA). Peptides (1 μg of each peptide mixture) were loaded into a trap column, washed for 6 min at a flow rate of 8 μL min^–1^, and separated at a flow rate of 250 nL min^–1^ using an 11 cm pico-frit chromatographic column packed in-house with C18 material, as described in Ebinezer et al. [77]. Peptides were eluted for 90 min using a 3% to 50% linear gradient of ACN/0.1% FA at a 250 nL min^–1^ flow rate. The instrument performed a full scan at high resolution (60,000) on the Orbitrap, followed by MS/MS scans on the three most intense ions with both CID (for identification) and HCD fragmentation (for quantification) performed on the same precursor.

Data were searched with Proteome Discoverer software (Thermo Fisher Scientific, Bremen, Germany), as described below; all peptides identified with two unique peptides and high (99%) confidence were used to generate a static excluding list. All samples were re-analyzed under identical instrumental and chromatographic conditions, except for the application of the excluded list.

### 4.6. LC-MS/MS Data Analysis

Raw MS/MS files were analyzed using Proteome Discoverer 1.4 (Thermo Fisher Scientific, Bremen, Germany) and searched using both Sequest HT (Thermo Fisher Scientific, Bremen, Germany) and the Mascot Search Engine (Matrix Science, London, UK) for protein identification. Data were filtered to exclude MS/MS spectra containing less than five peaks and with a total ion count lower than 50. Data were searched against the newly expanded and updated RNAseq database for *R. serbica* leaves (205,636 sequences) and concatenated with the sequences of the most common contaminants found in proteomics experiments.

Peptide and fragment tolerances were set to 10 ppm and 0.6 Da respectively. Enzyme specificity was set to trypsin with up to two missed cleavages. Carbamidomethyl cysteine and six-plex TMT labels at N-terminus and lysine residues were set as fixed modifications; oxidation of methionine was set as variable modification. The results obtained from both search engines were combined by Proteome Discoverer in a single output file. Percolator was used to calculate false discovery rates (FDR). Proteins were grouped into families according to the principle of maximum parsimony. Data were further filtered by considering as positive hits only the proteins that were identified with at least two unique peptides and quantified with at least two independent peptides.

Differentially abundant proteins were mapped into metabolic pathways using Mapman 3.6.0RC1 software [20]. The functional classification of 370 DAPs using the TAIR codes was performed manually.

Subcellular location prediction of identified DAPs was conducted using the TargetP1.1 server [78] and Protein Prowler Subcellular Localisation Predictor version 1.2 (available at: http://bioinf.scmb.uq.edu.au:8080/pprowler_webapp_1-2/, accessed on 23 March 2022). To predict the specific compartmentalization of each LEAP, the WoLF-PSORT tool was used (available at: https://wolfpsort.hgc.jp/, accessed on 23 March 2022). Protein-protein interactions studies were performed using STRING version 11.5 (available at: https://string-db.org/, accessed on 23 March 2022) to explore all the possible protein partners involved in associative functional interaction networks.

### 4.7. Fast Chlorophyll Fluorescence Induction Kinetics (OJIP) and Analysis of Photosynthetic Pigments

Measurements of OJIP in a direct imaging way were performed using 15 leaves from five different plants. Fully hydrated leaves were excised and placed in a measuring chamber as described by Küpper et al. [79]. Following five minutes of dark adaptation, the OJIP was measured using the same protocol as in Küpper et al. [79] in a FluorCam system (PSI, Brno, Czech Republic). All images were analyzed using the FluorCam 7 software (PSI, Brno, Czech Republic), as described by Küpper et al. [79]. The OJIP parameters describe the quantum yields of electron transport flux through PSII to PSI acceptors; Φ_Po_ = 1 − Fo/Fm, maximum quantum yield of primary PSII photochemistry; Φ_ET2o_ = 1 − Fj/Fm, the quantum yield of the electron transport flux from Q_A_ to Q_B_; Φ_RE1o_ = 1 − Fi/Fm quantum yield of the electron transport flux until the PSI electron acceptors; J^abs^/RC average absorbed photon flux per PSII reaction center.

Following the first measurement, ten leaves were placed in water (15 mL) flasks and allowed to slowly dry reaching about 57 ± 4% and 38 ± 8% RWC (five leaves each), and then OJIP was re-measured under the same conditions, while the remaining five leaves were kept in hydrated conditions. The leaves were kept in the growth chamber with a 16 h/8 h day/night photoperiod, 60% humidity, and 21–24 °C temperature. The maximal PAR intensity at noon was 200 μmol m^−2^ s^−1^ in a stepwise cycle.

Afterwards, the leaves were quickly frozen in liquid nitrogen, freeze-dried and 10–15 mg of powdered tissue was used for extraction of photosynthetic pigments using 100% acetone. The concentrations of chl *a*, chl *b*, and carotenoids were determined from the absorbances at 663 nm, 645 nm, and 470 nm following equations by Lichtenthaler and Wellburn [80] using a Lambda750 absorption spectrophotometer (Perkin–Elmer, Waltham, MA, USA).

### 4.8. Extraction and Measurement of Polyphenol Oxidases and Citrate Synthase Activities

The extraction of polyphenol oxidases (PPO) from *R. serbica* HL and DL was performed using a mortar and pestle with liquid nitrogen in the extraction buffer containing 50 mM TRIS-HCl pH 7.6, 1 mM EDTA, 5% polyvinylpyrrolidone (PVP), 5 mM ascorbate, and 1 mM phenylmethylsulphonyl fluoride (PMSF). The PPO activity was determined in 50 mM sodium phosphate buffer pH 5.5 following oxidation of 4 mM chlorogenic acid as an absorbance increase at 410 nm at 28 °C as described in Veljović-Jovanović [59].

The extraction medium for citrate synthase (CS) contained 50 mM TRIS-HCl pH 7.6, 1 mM EDTA, 5% PVP, 1% bovine serum albumin, 1 mM dithiocarbamate, 1 mM PMSF, 5 mM ascorbate, and 0.2% (*w*/*v*) Triton X-100. Extracts were obtained after centrifugation at 10,000× *g* at 4 °C). The activity of CS was assayed following this. The reaction mixture consisted of 100 mM TRIS-HCl pH 8, 0.25 mM 5,5′-dithiobis-(2-nitrobenzoic acid), 0.2 mM oxaloacetate, and 0.1 mM acetyl-CoA. The reaction started with the addition of 100 µL of crude extract and the absorbance increase at 412 nm was followed for 3 min at 25 °C (ε = 13,600 mM^−1^ cm^−1^). A unit of CS was defined as the amount of enzyme capable of forming 1 µmol of CoA-SH per minute under the described assay conditions. The protein contents in the samples were determined according to Bradford [76]. All spectrophotometric measurements were performed in triplicates at 25 °C using a temperature-controlled spectrophotometer (Shimadzu, UV-160, Kyoto, Japan).

### 4.9. Phenolics Analysis

Frozen HL and DL were rapidly homogenized in liquid nitrogen, extracted in methanol containing 0.1% HCl, followed by acidic hydrolysis for aglycone determination as previously described in Vidović et al. [54]. Extractions were performed in duplicates and finally flushed with nitrogen.

Analyses were performed by HPLC coupled with a photodiode array detector (LC-20AB Prominence liquid chromatograph, Shimadzu, Kyoto, Japan) on 250 × 4.6 mm, 5.0 mm, Luna C18 (2) reversed-phase column (Phenomenex Ltd. Torrance, CA, USA). The elution conditions were exactly as described previously [54]. Specific phenolic compounds were identified by comparing the absorption spectra to authentic standards and by spiking. Quantification was performed by peak area using Shimadzu LC Solution software (Shimadzu, Kyoto, Japan).

### 4.10. Soluble Sugar Analysis

The content of soluble carbohydrates was measured according to Vidović et al. [54]. Approximately 0.1 g of frozen leaf tissue was homogenized in liquid nitrogen, extracted in 1 mL of ultrapure water (1/10, *w*/*v*), and placed in an ultrasonic bath at room temperature for 30 min. Samples were centrifuged at 14,000× *g* for 10 min at 4 °C. The pellet was re-extracted with ultrapure methanol in the same manner as previously described with water. The obtained supernatants were pooled together for further analysis.

Chromatographic separations were performed using a DIONEX ICS 3000 DP liquid chromatography system (Dionex, Sunnyvale, CA, USA) equipped with a quaternary gradient pump (Dionex, Sunnyvale, CA, USA). The carbohydrates were separated on a CarboPac^®^ PA100 pellicular anion–exchange column (4 × 250 mm) (Dionex, Sunnyvale, CA, USA) at 30 °C. The elution gradient and detection were the same as described in Vidović et al. [54].

### 4.11. Cell Wall Isolation and Purification

The plant cell wall was isolated according to the procedure described in Živanović et al. [22]. A series of organic solvents extractions were performed to remove pigments, alkaloids, tannins, soluble sugars, and other low molecular weight metabolites from the cell wall containing residues. Leaves were powdered with a mortar and pestle in liquid nitrogen and extracted in 80% methanol (1/8, *w*/*v*) with shaking for 60 min at room temperature. The homogenate was centrifuged at 1000× *g* for 20 min at room temperature and the pellet was washed two times with 80% methanol. The pellet was resuspended in 1 M NaCl with 0.5% Triton X-100 and centrifuged at 1000× *g* for 20 min at room temperature. The pellet was rinsed with distilled water, once with absolute methanol and twice with acetone. The purified cell wall was dried and used for structural analyses.

### 4.12. Infrared (IR) Spectroscopy of Cell Wall Fractions

Fourier transform IR (FTIR) spectroscopy was used to profile the HL and DL cell walls. The methodology offers a rapid non-invasive and non-destructive method to obtain data and provide insight into the underlying chemical nature of the analyzed material. The FTIR spectra of the extracted cell wall materials were recorded using a Perkin Elmer Spectrum Two spectrometer equipped with the Universal ATR accessory. The spectrum of each powder sample was collected in the range of 4000–400 cm^−1^ with 200 scans and a spectral resolution of 4 cm^−1^. The obtained spectral data presented superposition of spectral signatures from the carbohydrate and phenolic cell wall polymers, and identification of the various chemical functional groups (and the polymers that contain them) was performed through comparison with reference data [22,23]. Baseline correction was performed using the trial Spectra Gryph software (available at: https://www.effemm2.de/spectragryph/index.html, accessed on 10 March 2022).

### 4.13. Phylogenetic Analysis and Conserved Motif Composition of R. serbica GLPs 

Multiple sequence alignment (MSA) of the full-length sequences of *R. serbica* GLP proteins was performed using the MAFFT v7 (available at: https://mafft.cbrc.jp/alignment/server/, accessed on 23 March 2022), L-INS-i method with 1000 iterations of improvement, the BLOSUM62 scoring matrix, and a gap opening penalty of 1.53. A phylogenetic tree was created using the EMBL-EBI Simple Phylogeny tool (available at: https://www.ebi.ac.uk/Tools/phylogeny/simple_phylogeny/, accessed on 23 March 2022) with the neighbor-joining method and default parameters. iTOL—Interactive Tree of Life v.6.5 (available at: https://itol.embl.de/, accessed on 23 March 2022) was used to display and annotate the tree.

The Multiple Expectation Maximization for Motif EliCitation (MEME) online tool (available at: https://meme-suite.org/meme/tools/meme, accessed on 23 March 2022) was used to identify the conserved protein motifs. MEME was run using the “zero or one occurrence per sequence” mode and searched for five different motifs with a minimum width value of 6 and a maximum width of 50. All other parameters were left at their default values. Obtained MEME outputs (in XML format) were exported into interactive iTOL online software to couple and visualize motifs with the phylogenetic tree of each LEA protein family group, separately.

### 4.14. Statistics

For proteomic analysis, four biological replicates for each group were used. To test the significant differences in phenolic compound, sugar, and chlorophyll contents, as well as chlorophyll fluorescence parameters between HL and DL, Mann–Whitney U/*t*-test was used, and the significance threshold value was set at 0.05.

## 5. Conclusions

This paper presents a comprehensive study of the gene and protein expression profiles, as well as changes in the cell wall, soluble carbohydrates and phenolics metabolism in response to desiccation. Desiccation tolerance in *R. serbica* is a species-specific process orchestrated by several metabolic pathways and regulatory networks acting at several levels:

(i) shutting down linear PET and switching on CET at PS I, together with accumulation of PPOs and OEEs might be responsible for decreasing H_2_O_2_/O_2_^●–^ formation, either via oxygen buffering or PTM-based regulation;

(ii) accumulation of enzymatic H_2_O_2_ producers in chloroplasts (Cu/Zn SOD) and the cell wall (GLPs), and reducing the levels of H_2_O_2_ scavengers (the Asc-GSH cycle), PODs, and CATs;

(iii) remodeling of the cell wall composition (polymer content and demethylation degree), and cell wall loosening that might be HO^●^-dependent and POD-independent;

(iv) recycling amino acids from highly abundant photosynthesis- and structural-cell-wall-related proteins to LEA4 proteins and dehydrins able to stabilize proteins, membranes, and nucleic acids, by molecular shielding or liquid-liquid phase separation;

(v) dynamic carbon recycling from starch to other sugar osmolytes.

Taken together, the physiological state of *R. serbica* during desiccation is similar to dormancy, when, instead of investing energy and photosynthates in growth, metabolism is switched to the protection of cellular constituents until optimal water conditions return.

## Figures and Tables

**Table 1:** Content of soluble sugars in HL and DL of *R. serbica*. Values are presented as means ± SE (*n* = 5–6). Asterisks denote significant differences between treatments and respective controls according to the *t*-test (* *p* < 0.05, ** *p* < 0.01).

	HL	DL		HL	DL
Hexose (mmol g^−1^ DW)			Oligosaccharides (µmol g^−1^ DW)		
Glucose	0.94 ± 0.15	2.26 ± 0.34 **	Isomaltotriose	2.7 ± 0.6	7.8 ± 2.7
Fructose	0.71 ± 0.10	2.06 ± 0.40 **	Maltotriose	6.3 ± 1.5	6.7 ± 1.7
Galactose	0.19 ± 0.02	0.79 ± 0.10 *	Raffinose	6.4 ± 2.9	16.4 ± 2.8 *
Pentose (µmol g^−1^ DW)			Melezitose	2.9 ± 1.2	10.0 ± 2.0 *
Arabinose	64.4 ± 19.9	168.6 ± 57.2	Panose	0.23 ± 0.12	0.82 ± 0.32
Ribose	26.5 ± 6.5	105.3 ± 21.1 *	Stachyose	0.70 ± 0.21	4.54 ± 0.81 **
Rhamnose	2.6 ± 0.4	25.1 ± 5.6 *	Sugar alcohols (µmol g^−1^ DW)		
Xylose	12.3 ± 3.3	34.7 ± 6.9 *	Erythritol	75.23 ± 27.5	213.7 ± 50.5 *
Disaccharides (µmol g^−1^ DW)			Sorbitol	48.9 ± 16.2	155.0 ± 60.7
Sucrose	317.4 ± 60.1	1367.5 ± 461.3	Galactitol	122.0 ± 41.7	296.9 ± 171.5
Trehalose	29.2 ± 4.7	79.1 ± 31.8	Arabinitol	12.3 ± 4.6	66.5 ± 18.4 *
Melibiose	2.4 ± 0.7	5.7 ± 1.9	Mannitol	6.2 ± 1.2	23.5 ± 8.8
Maltose	13.6 ± 4.2	62.4 ± 18.9 *			
Isomaltose	2.7 ± 0.6	7.8 ± 2.7 *	Total soluble sugars (mmol g^−1^ DW)		
Turanose	90.4 ± 28.0	312.2 ± 76.6 *		2.7 ± 0.3	8.2 ± 1.0 **

**Table 2:** The endogenous citrate synthase (CS) and polyphenol oxidase (PPO) in vitro activity during *R. serbica* desiccation. Values represent mean ± SE, *n* = 3–5. Asterisks denote significant differences between treatments and respective controls according to *t*-test (* *p* < 0.05; ** *p* > 0.01).

Treatment	CS, U g^–1^_DW_	PPO, ΔA_410_ mg^–1^_prot_
HL (90% RWC)	0.72 ± 0.08	52.3 ± 4.7
sDL (50% RWC)	0.95 ± 0.03	84.9 ± 6.8 **
DL (15–20% RWC)	1.07 ± 0.04 *	142.6 ± 3.0 **

**Table 3:** Differentially abundant LEAPs in *R. serbica* upon desiccation.

LEAP Family Subgroup	Rs_code	Fold Change (DL/HL)	Subcellular Localisation *
LEA1.3	Rs_170082	1.6	nucleus
LEA2.2	Rs_151841	−1.3	nucleus
LEA2.2	Rs_70056	−1.4	chloroplasts; cytosol
LEA2.2	Rs_70057	−1.6	cytosol
LEA4.3	Rs_139539	2.3	chloroplasts; mitochondrion
LEA4 **	Rs_191445	2.3	mitochondrion
LEA4.3	Rs_67538	2.1	chloroplast
LEA4 **	Rs_121834	2.1	mitochondrion
LEA4.3	Rs_136891	2.0	nucleus
LEA4.3	Rs_3451	2.0	chloroplast
LEA4.3	Rs_67537	1.9	nucleus
LEA4.3	Rs_67210	1.9	nucleus, mitochondrion
LEA4.3	Rs_67211	1.7	mitochondrion
LEA4.3	Rs_67212	1.7	mitochondrion
LEA4.3	Rs_67539	1.6	chloroplast
LEA4.3	Rs_205247	1.6	chloroplast
LEA4.3	Rs_205105	1.6	chloroplast
LEA4.3	Rs_52085	1.5	mitochondrion
LEA4.3	Rs_140058	1.4	nucleus
LEA4 **	Rs_187459	1.4	mitochondrion, nucleus
Other (LEA4)	Rs_67929	1.4	cytosol
LEA4.2	Rs_108587	1.4	nucleus, mitochondrion
LEA4 **	Rs_165950	1.3	nucleus
DEH **	Rs_194906	1.3	nucleus
DEH1	Rs_107018	1.3	nucleus, mitochondrion
SMP	Rs_104365	−1.6	nucleus; cytosol

* Predicted subcellular distribution. ** For LEAPs containing less than 100 aa, a subgroup was not estimated.

## Supplementary Materials

The following supporting information can be downloaded at: https://www.mdpi.com/article/10.3390/plants11091199/s1, Figure S1. GO of 9578 identified DEGs and 370 identified DAPs in *R. serbica* HL and DL; Figure S2. Graphical representation of PPI network analysis of DAPs involved in antioxidative metabolism in *R. serbica* HL and DL; Figure S3. MEME motifs and motif logos of *Ramonda serbica* germin-like proteins visualized in iTOL; Table S1. List of DEGs identified in *R. serbica* HL and DL; Table S2. List of DAPs identified in *R. serbica* HL and DL; Table S3. Proteomics data; Table S4. GO, KEGG and metabolic pathways of DEGs identified in *R. serbica* HL and DL; Table S5. Mapman mapping of DAPs identified in *R. serbica* HL and DL; Table S6. Mol % of soluble sugars in HL and DL of *R. serbica*; Table S7. Content of phenolics in methanol extracts of *R. serbica* HL and DL.

## References

[1] Rabara R.C., Tripathi P., Reese R.N., Rushton D.L., Alexander D., Timko M.P., Rushton P.J. (2015). Tobacco drought stress responses reveal new targets for *Solanaceae* crop improvement. BMC Genom..

[2] Farrant J.M., Hilhorst H.W.M. (2021). What is dry? Exploring metabolism and molecular mobility at extremely low water contents. J. Exp. Bot..

[3] Challabathula D., Zhang Q., Bartels D. (2018). Protection of photosynthesis in desiccation-tolerant resurrection plants. J. Plant Physiol..

[4] Scott P. (2000). Resurrection plants and the secrets of eternal leaf. Ann. Bot..

[5] Veljović-Jovanović S., Kukavica B., Stevanović B., Navari-Izzo F. (2006). Senescence-and drought-related changes in peroxidase and superoxide dismutase isoforms in leaves of *Ramonda serbica*. J. Exp. Bot..

[6] Challabathula D., Bartels D. (2013). Desiccation tolerance in resurrection plants: New insights from transcriptome, proteome and metabolome analysis. Front. Plant Sci..

[7] Liu J., Moyankova D., Lin C.T., Mladenov P., Sun R.Z., Djilianov D., Deng X. (2018). Transcriptome reprogramming during severe dehydration contributes to physiological and metabolic changes in the resurrection plant *Haberlea rhodopensis*. BMC Plant Biol..

[8] Farrant J.M., Moore J.P. (2011). Programming desiccation-tolerance: From plants to seeds to resurrection plants. Curr. Opin. Plant Biol..

[9] Chandra J., Keshavkant S. (2018). Desiccation-induced ROS accumulation and lipid catabolism in recalcitrant. Physiol. Mol. Biol. Plants.

[10] Gechev T.S., Dinakar C., Benina M., Toneva V., Bartels D. (2012). Molecular mechanisms of desiccation tolerance in resurrection plants. Cell. Mol. Life Sci..

[11] Bartels D. (2005). Desiccation tolerance studied in the resurrection plant *Craterostigma plantagineum*. Integr. Comp. Biol..

[12] Olvera-Carrillo Y., Campos F., Reyes J.L., Garciarrubio A., Covarrubias A.A. (2010). Functional analysis of the group 4 late embryogenesis abundant proteins reveals their relevance in the adaptive response during water deficit in Arabidopsis. Plant Physiol..

[13] Farrant J.M. (2007). Mechanisms of desiccation tolerance in angiosperm resurrection plants. Plant Stress.

[14] Strasser R.J., Tsimilli-Michael M., Qiang S., Goltsev V. (2010). Simultaneous in vivo recording of prompt and delayed fluorescence and 820-nm reflection changes during drying and after rehydration of the resurrection plant *Haberlea rhodopensis*. Biochim. Biophys. Acta–Bioenerg..

[15] Dirk L.M.A., Abdel C.G., Ahmad I., Neta I.C.S., Pereira C.C., Pereira F.E.C.B., Unêda-Trevisoli S.H., Pinheiro D.G., Downie A.B. (2020). Late embryogenesis abundant protein-client protein interactions. Plants.

[16] Ginsawaeng O., Heise C., Sangwan R., Karcher D., Hernández-Sánchez I.E., Sampathkumar A., Zuther E. (2021). Subcellular localization of seed-expressed LEA_4 proteins reveals liquid-liquid phase separation for LEA9 and for LEA48 homo- and LEA42-LEA48 heterodimers. Biomolecules.

[17] Dražić G., Mihailović N., Stevanović B. (1999). Chlorophyll metabolism in leaves of higher poikilohydric plants *Ramonda serbica* Panč. and *Ramonda nathaliae* Panč. et Petrov. during dehydration and rehydration. J. Plant Physiol..

[18] Zhu Y., Wang B., Phillips J., Zhang Z.N., Du H., Xu T., Huang L.C., Zhang X.F., Xu G.H., Li W.L. (2015). Global transcriptome analysis reveals acclimation–primed processes involved in the acquisition of desiccation tolerance in *Boea hygrometrica*. Plant Cell Physiol..

[19] Artur M.A.S., Zhao T., Ligterink W., Schranz E., Hilhorst H.W.M. (2019). Dissecting the genomic diversification of late embryogenesis abundant (LEA) protein gene families in plants. Genome Biol. Evol..

[20] Thimm O., Bläsing O., Gibon Y., Nagel A., Meyer S., Krüger P., Stitt M. (2004). MAPMAN: A user-driven tool to display genomics data sets onto diagrams of metabolic pathways and other biological processes. Plant J..

[21] Pantelić A., Stevanović S., Komić S.M., Kilibarda N., Vidović M. (2022). In silico characterisation of the late embryogenesis abundant (LEA) protein families and their role in desiccation tolerance in *Ramonda serbica* Panc. Int. J. Mol. Sci..

[22] Živanović B., Milić-Komić S., Nikolić N., Mutavdžić D., Srećković T., Veljović-Jovanović S., Prokić L. (2021). Differential response of two tomato genotypes, wild type cv. Ailsa Craig and its ABA-deficient mutant flacca to short-termed drought cycles. Plants.

[23] Alonso-Simón A., García-Angulo P., Mélida H., Encina A., Álvarez J.M., Acebes J.L. (2011). The use of FTIR spectroscopy to monitor modifications in plant cell wall architecture caused by cellulose biosynthesis inhibitors. Plant Signal. Behav..

[24] Ross A.B., Langer J.D., Jovanovic M. (2021). Proteome turnover in the spotlight: Approaches, applications, and perspectives. Mol. Cell. Proteom..

[25] Vogel C., Marcotte E.M. (2012). Insights into the regulation of protein abundance from proteomic and transcriptomic analyses. Nat. Rev. Genet..

[26] Fernie A.R., Stitt M. (2012). On the discordance of metabolomics with proteomics and transcriptomics: Coping with increasing complexity in logic, chemistry, and network interactions scientific correspondence. Plant Physiol..

[27] Liang C., Cheng S., Zhang Y., Sun Y., Fernie A.R., Kang K., Panagiotou G., Lo C., Lim B.L. (2016). Transcriptomic, proteomic and metabolic changes in *Arabidopsis thaliana* leaves after the onset of illumination. BMC Plant Biol..

[28] Xu X., Legay S., Sergeant K., Zorzan S., Leclercq C.C., Charton S., Giarola V., Liu X., Challabathula D., Renaut J. (2021). Molecular insights into plant desiccation tolerance: Transcriptomics, proteomics and targeted metabolite profiling in *Craterostigma plantagineum*. Plant J..

[29] Rakić T., Lazarević M., Jovanović Z.S., Radović S., Siljak–Yakovlev S., Stevanović B., Stevanović V. (2014). Resurrection plants of the genus Ramonda: Prospective survival strategies—Unlock further capacity of adaptation, or embark on the path of evolution?. Front. Plant Sci..

[30] Moore J.P., Nguema-Ona E.E., Vicré-Gibouin M., Sørensen I., Willats W.G., Driouich A., Farrant J.M. (2013). Arabinose-rich polymers as an evolutionary strategy to plasticize resurrection plant cell walls against desiccation. Planta.

[31] Jung N.U. (2020). Molecular and Biochemical Studies of the *Craterostigma plantagineum* Cell Wall during Dehydration and Rehydration. Ph.D. Thesis.

[32] Vicré M., Lerouxel O., Farrant J., Lerouge P., Driouich A. (2004). Composition and desiccation-induced alterations of the cell wall in the resurrection plant *Craterostigma wilmsii*. Physiol. Plant..

[33] Wormit A., Usadel B. (2018). The multifaceted role of pectin methylesterase inhibitors (PMEIs). Int. J. Mol. Sci..

[34] Jung N.U., Giarola V., Chen P., Knox J.P., Bartels D. (2019). *Craterostigma plantagineum* cell wall composition is remodelled during desiccation and the glycine-rich protein CpGRP1 interacts with pectins through clustered arginines. Plant J..

[35] Levesque-Tremblay G., Pelloux J., Braybrook S.A., Müller K. (2015). Tuning of pectin methylesterification: Consequences for cell wall biomechanics and development. Planta.

[36] Wang L., Shang H., Liu Y., Zheng M., Wu R., Phillips J., Bartels D., Deng X. (2009). A role for a cell wall localized glycine-rich protein in dehydration and rehydration of the resurrection plant *Boea hygrometrica*. Plant Biol..

[37] Giarola V., Krey S., von den Driesch B., Bartels D. (2016). The *Craterostigma plantagineum* glycine-rich protein CpGRP1 interacts with a cell wall-associated protein kinase 1 (CpWAK1) and accumulates in leaf cell walls during dehydration. New Phytol..

[38] Pei Y., Li X., Zhu Y., Ge X., Sun Y., Liu N., Jia Y., Li F., Hou Y. (2019). GhABP19, a novel germin-like protein from *Gossypium hirsutum*, plays an important role in the regulation of resistance to *Verticillium* and *Fusarium* wilt pathogens. Front. Plant Sci..

[39] Veljović-Jovanović S., Kukavica B., Vidović M., Morina F., Menckhoff L., Gupta D.K., Palma J.M., Corpas F.J. (2018). Class III peroxidases: Functions, localization and redox regulation of isoenzymes. Antioxidants and Antioxidant Enzymes in Higher Plants.

[40] Pristov J.B., Mitrović A., Spasojević I. (2011). A comparative study of antioxidative activities of cell-wall polysaccharides. Carbohydr. Res..

[41] Kukavica B., Mojović M., Vučinić Ž., Maksimović V., Takahama U., Veljović-Jovanović S. (2009). Generation of hydroxyl radical in isolated pea root cell wall, and the role of cell wall-bound peroxidase, Mn-SOD and phenolics in their production. Plant Cell Physiol..

[42] Collett H.M., Butowt R., Smith J., Farrant J., Illing N. (2003). Photosynthetic genes are differentially transcribed during the dehydration-rehydration cycle in the resurrection plant, *Xerophyta humilis*. J. Exp. Bot..

[43] Yang E.J., Oh Y.A., Lee E.S., Park A.R., Cho S.K., Yoo Y.J., Park O.K. (2003). Oxygen-evolving enhancer protein 2 is phosphorylated by glycine-rich protein 3/wall-associated kinase 1 in Arabidopsis. Biochem. Biophys. Res. Commun..

[44] Jiang G., Wang Z., Shang H., Yang W., Hu Z., Phillips J., Deng X. (2007). Proteome analysis of leaves from the resurrection plant *Boea hygrometrica* in response to dehydration and rehydration. Planta.

[45] Farrant J.M. (2000). A comparison of mechanisms of desiccation tolerance among three angiosperm resurrection plant species. Plant Ecol..

[46] Heber U., Bilger W., Bligny R., Lange O.L. (2000). Photo-tolerance of lichens, mosses and higher plants in an alpine environment: Analysis of photoreactions. Planta.

[47] Huang W., Yang S.-J., Zhang S.-B., Zhang J.-L., Cao K.-F. (2012). Cyclic electron flow plays an important role in photoprotection for the resurrection plant *Paraboea rufescens* under drought stress. Planta.

[48] Tan T., Sun Y., Luo S., Zhang C., Zhou H., Lin H. (2017). Efficient modulation of photosynthetic apparatus confers desiccation tolerance in the resurrection plant *Boea hygrometrica*. Plant Cell Physiol..

[49] Mladenov P., Finazzi G., Bligny R., Moyankova D., Zasheva D., Boisson A.-M., Brugière S., Krasteva V., Alipieva K., Simova S. (2015). In vivo spectroscopy and NMR metabolite fingerprinting approaches to connect the dynamics of photosynthetic and metabolic phenotypes in resurrection plant Haberlea rhodopensis during desiccation and recovery. Front. Plant Sci..

[50] Markovska Y., Tsonev T., Kimenov G. (1997). Regulation of cam and respiratory recycling by water supply in higher poikilohydric plants—*Haberlea rhodopensis* Friv. and *Ramonda serbica* Panc, at transition from biosis to anabiosis and vice versa. Bot. Acta.

[51] Kirch H.H., Nair A., Bartels D. (2001). Novel ABA-and dehydration-inducible aldehyde dehydrogenase genes isolated from the resurrection plant *Craterostigma plantagineum* and *Arabidopsis thaliana*. Plant J..

[52] Živković T., Quartacci M.F., Stevanović B., Marinone F., Navari-Izzo F. (2005). Low molecular weight substances in the poikilohydric plant Ramonda serbica during dehydration and rehydration. Plant Sci..

[53] Ingram J., Bartels D. (1996). The molecular basis of dehydration tolerance in plants. Annu. Rev. Plant Physiol..

[54] Vidović M., Morina F., Milić S., Albert A., Zechmann B., Tosti T., Winkler J.B., Jovanović S.V. (2015). Carbon allocation from source to sink leaf tissue in relation to flavonoid biosynthesis in variegated *Pelargonium zonale* under UV-B radiation and high PAR intensity. Plant Physiol. Biochem..

[55] Nishizawa A., Yabuta Y., Shigeoka S. (2008). Galactinol and raffinose constitute a novel function to protect plants from oxidative damage. Plant Physiol..

[56] Dietz K.J., Jacob S., Oelze M.L., Laxa M., Tognetti V., de Miranda S.M., Baier M., Finkemeier I. (2016). The function of peroxiredoxins in plant organelle redox metabolism. J. Exp. Bot..

[57] Augusti A., Scartazza A., Navari-Izzo F., Sgherri C.L.M., Stevanović B., Brugnoli E. (2001). Photosystem II photochemical efficiency, zeaxanthin and antioxidant contents in the poikilohydric *Ramonda serbica* during dehydration and rehydration. Photosynth. Res..

[58] Vidović M., Franchin C., Morina F., Veljović-Jovanović S., Masi A., Arrigoni G. (2020). Efficient protein extraction for shotgun proteomics from hydrated and desiccated leaves of resurrection *Ramonda serbica* plants. Anal. Bioanal. Chem..

[59] Vidović M., Morina F., Veljović-Jovanović S., Singh V.P., Singh S., Prasad S.M., Parihar P. (2017). Stimulation of various phenolics in plants under ambient UV-B radiation. UV-B Radiation: From Environmental Stressor to Regulator of Plant Growth.

[60] Veljović-Jovanović S., Kukavica B., Navari-Izzo F. (2008). Characterization of polyphenol oxidase changes induced by desiccation of *Ramonda serbica* leaves. Physiol. Plant..

[61] Sgherri C., Stevanovic B., Navari-Izzo F. (2004). Role of phenolics in the antioxidative status of the resurrection plant *Ramonda serbica* during dehydration and rehydration. Physiol. Plant..

[62] Takahama U. (2004). Oxidation of vacuolar and apoplastic phenolic substrates by peroxidase: Physiological significance of the oxidation reactions. Phytochem. Rev..

[63] Boeckx T., Winters A.L., Webb K.J., Kingston-Smith A.H. (2015). Polyphenol oxidase in leaves: Is there any significance to the chloroplastic localization?. J. Exp. Bot..

[64] Bremer A., Wolff M., Thalhammer A., Hincha D.K. (2017). Folding of intrinsically disordered plant LEA proteins is driven by glycerol-induced crowding and the presence of membranes. FEBS J..

[65] Cuevas-Velazquez C.L., Reyes J.L., Covarrubias A.A. (2017). Group 4 late embryogenesis abundant proteins as a model to study intrinsically disordered proteins in plants. Plant Signal. Behav..

[66] Candat A., Paszkiewicz G., Neveu M., Gautier R., Logan D.C., Avelange-Macherel M.H., Macherel D. (2014). The ubiquitous distribution of late embryogenesis abundant proteins across cell compartments in Arabidopsis offers tailored protection against abiotic stress. Plant Cell.

[67] Koag M.C., Wilkens S., Fenton R.D., Resnik J., Vo E., Close T.J. (2009). The K-segment of maize DHN1 mediates binding to anionic phospholipid vesicles and concomitant structural changes. Plant Physiol..

[68] Hara M., Shinoda Y., Tanaka Y., Kuboi T. (2009). DNA binding of citrus dehydrin promoted by zinc ion. Plant Cell Environ..

[69] Liu X., Wang Z., Wang L., Wu R., Phillips J., Deng X. (2009). LEA 4 group genes from the resurrection plant *Boea hygrometrica* confer dehydration tolerance in transgenic tobacco. Plant Sci..

[70] Olvera-Carrillo Y., Luis Reyes J., Covarrubias A.A. (2011). Late embryogenesis abundant proteins: Versatile players in the plant adaptation to water limiting environments. Plant Signal. Behav..

[71] Chakrabortee S., Tripathi R., Watson M., Schierle G.S., Kurniawan D.P., Kaminski C.F., Wise M.J., Tunnacliffe A. (2012). Intrinsically disordered proteins as molecular shields. Mol. Biosyst..

[72] Belott C., Janis B., Menze M.A. (2020). Liquid-liquid phase separation promotes animal desiccation tolerance. Proc. Natl. Acad. Sci. USA.

[73] Rohrig H., Schmidt J., Colby T., Brautigam A., Hufnagel P., Bartels D. (2006). Desiccation of the resurrection plant *Craterostigma plantagineum* induces dynamic changes in protein phosphorylation. Plant Cell Environ..

[74] Biundo A., Braunschmid V., Pretzler M., Kampatsikas I., Darnhofer B., Birner-Gruenberger R., Rompel A., Ribitsch D., Guebitz G.M. (2020). Polyphenol oxidases exhibit promiscuous proteolytic activity. Commun. Chem..

[75] Harten J.B., Eickmeier W.G. (1986). Enzyme dynamics of the resurrection plant *Selaginella lepidophylla* (Hook. & Grev.) spring during rehydration. Plant Phys..

[76] Bradford M. (1976). A rapid and sensitive method for the quantitation of microgram quantities of protein utilizing the principle of protein-dye binding. Anal. Biochem..

[77] Ebinezer L.B., Franchin C., Trentin A.R., Carletti P., Trevisan S., Agrawal G.K., Quaggiotti S., Arrigoni G., Masi A. (2020). Quantitative proteomics of maize roots treated with a protein hydrolysate: A comparative study with transcriptomics highlights the molecular mechanisms responsive to biostimulants. J. Agric. Food Chem..

[78] Emanuelsson O., Brunak S., von Heijne G., Nielsen H. (2007). Locating proteins in the cell using TargetP, SignalP and related tools. Nat. Protoc..

[79] Küpper H., Benedikty Z., Morina F., Andresen E., Mishra A., Trtilek M. (2019). Analysis of OJIP chlorophyll fluorescence kinetics and QA reoxidation kinetics by direct fast imaging. Plant Physiol..

[80] Lichtenthaler H.K., Wellburn A.R. (1983). Determinations of total carotenoids and chlorophylls a and b of leaf extracts in different solvents. Biochem. Soc. Trans..

