# Supplementary material for: Desiccation Tolerance in Ramonda serbica Panc.: An Integrative Transcriptomic, Proteomic, Metabolite and Photosynthetic Study
Source: Plants (Basel). 2022 Apr 28;11(9):1199. doi: 10.3390/plants11091199 (PMC9104375; doi:10.3390/plants11091199)
Supplement: Supplementary file 1 [file plants-11-01199-s001.zip › Supplementary Figure S1.pdf]

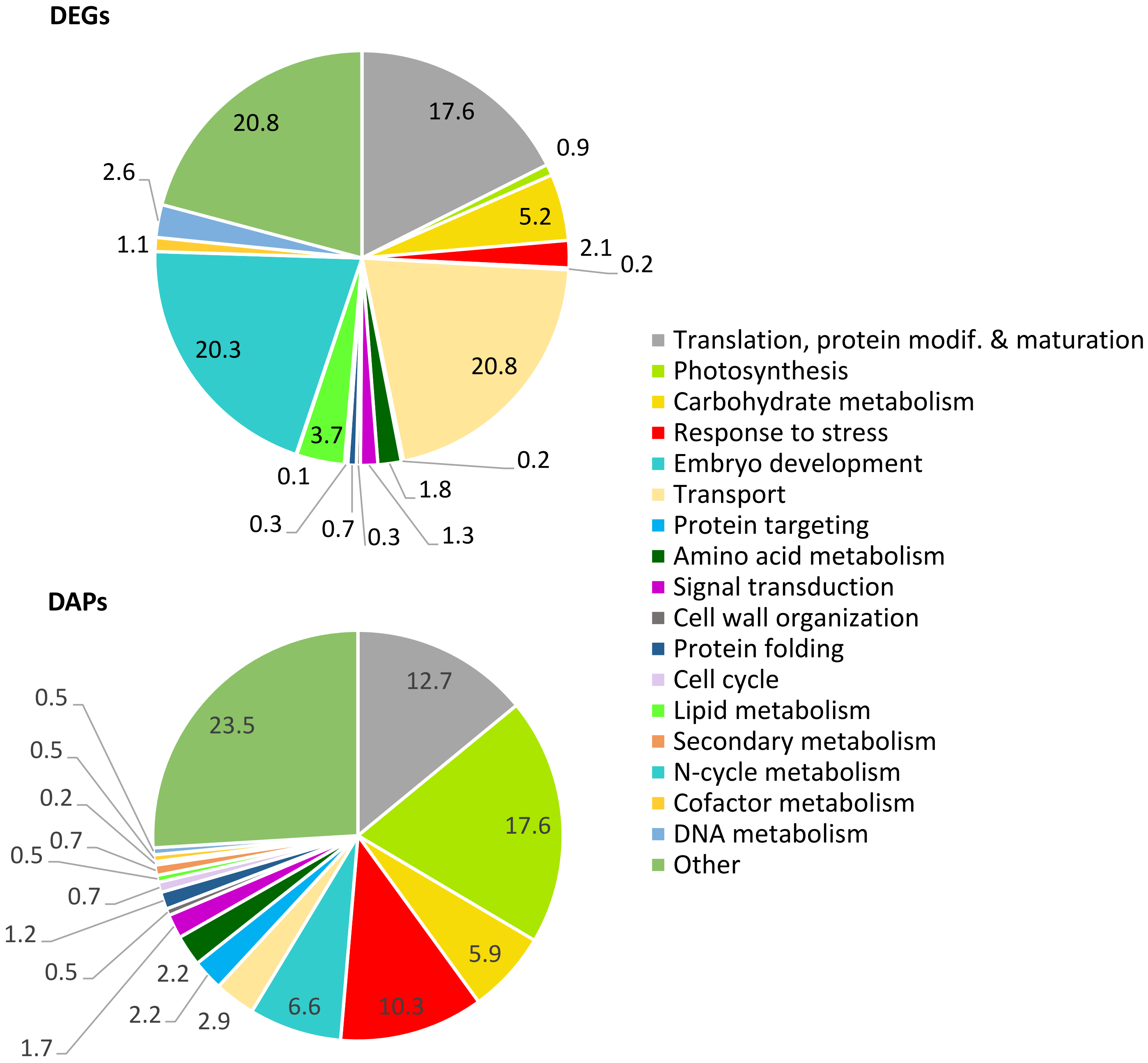
DEGs
17.6
0.9
5.2
2.1
0.2
20.8
0.2
1.8
1.3
0.3
0.7
0.3
0.1
3.7
20.3
1.1
2.6
20.8
DAPs
12.7
17.6
5.9
10.3
6.6
2.9
2.2
2.2
1.7
0.5
1.2
0.7
0.5
0.7
0.2
0.5
0.5
23.5
Translation, protein modif. & maturation
Photosynthesis
Carbohydrate metabolism
Response to stress
Embryo development
Transport
Protein targeting
Amino acid metabolism
Signal transduction
Cell wall organization
Protein folding
Cell cycle
Lipid metabolism
Secondary metabolism
N-cycle metabolism
Cofactor metabolism
DNA metabolism
Other
