# Supplementary material for: Desiccation Tolerance in Ramonda serbica Panc.: An Integrative Transcriptomic, Proteomic, Metabolite and Photosynthetic Study
Source: Plants (Basel). 2022 Apr 28;11(9):1199. doi: 10.3390/plants11091199 (PMC9104375; doi:10.3390/plants11091199)
Supplement: Supplementary file 1 [file plants-11-01199-s001.zip › Supplementary Table S6.pdf]

Supplementary Table S6. Mol % of soluble sugars in HL and DL of *R. serbica*. Values are presented as means ± SE (n = 5-6). Asterisks denote significant differences between treatments and respective controls according to the *t*-test (*P< 0.05, **P< 0.01, ***P< 0.001).

	HL	DL		HL	DL
Hexose			***Oligosaccharides***		
Glucose	33.7 ± 2.8	27.8 ± 3.0	Isomaltotriose	0.14 ± 0.05	4.19 ± 0.04
Fructose	25.6 ± 2.1	24.5 ± 2.9	Maltotriose	0.25 ± 0.05	2.55 ± 0.02
Galactose	7.2 ± 0.8	9.9 ± 1.1	Raffinose	0.23 ± 0.09	5.10 ± 0.02
Pentose			Melezitose	0.10 ± 0.03	4.58 ± 0.01
Arabinose	2.5 ± 0.8	5.9 ± 0.7	Panose	0.01 ± 0.01	5.64 ± 0.01
Ribose	0.9 ± 0.2	3.1 ± 0.3	Stachyose	0.02 ± 0.01	3.47 ± 0.01 ***
Rhamnose	0.09 ± 0.02	3.63 ± 0.4 **	***Sugar alcohols***		
Xylose	0.5 ± 0.2	4.0± 0.1	Erythritol	3.3 ± 1.1	6.3 ± 0.5
Disaccharides			Sorbitol	1.5 ± 0.5	5.4 ± 1.0
Sucrose	14.0 ± 4.8	16.5 ± 3.4	Galactitol	3.7 ± 1.2	7.5 ± 2.7
Trehalose	1.1 ± 0.1	2.2 ± 0.4	Arabinitol	0.57 ± 0.19	4.92 ± 0.27
Melibiose	0.11 ± 0.03	4.12 ± 0.03	mannitol	0.33 ± 0.10	3.9 ± 0.1
Maltose	0.53 ± 0.15	4.12 ± 0.26 *			
Isomaltose	1.4 ± 0.3	4.1 ± 0.3			
Turanose	3.3 ± 0.1	6.9 ± 1.2			
