# Supplementary material for: Desiccation Tolerance in Ramonda serbica Panc.: An Integrative Transcriptomic, Proteomic, Metabolite and Photosynthetic Study
Source: Plants (Basel). 2022 Apr 28;11(9):1199. doi: 10.3390/plants11091199 (PMC9104375; doi:10.3390/plants11091199)
Supplement: Supplementary file 1 [file plants-11-01199-s001.zip › Supplementary Table S7.pdf]

Supplementary Table S7. Content of phenolics in methanol extracts of *R. serbica* HL and DL. Values are given in μmol gDW^{-1} and presented as means ± SE (n = 3-5). Asterisks denote significant differences between treatments and respective controls according to *t*-test (*$P< 0.05$).

Phenolic compound	HL	DL
Syringic acid	22.4 ± 3.5	38.6 ± 8.1
Caffeic acid (CA)	4.82 ± 0.31	5.59 ± 0.70
Chlorogenic acid	5.40 ± 0.31	5.98 ± 0.89
p-coumaric acid	0.94 ± 0.16	0.94 ± 0.17
X337 (calc. as CA)	36.3 ± 3.0	45.6 ± 5.2 *
Catechin	126.6 ± 19.6	196.7 ± 37.4
Epicatechin	40.6 ± 4.2	39.1 ± 1.3
Quercetin	15.9 ± 1.6	15.4 ± 1.5
Kaempferol	14.5 ± 3.5	29.0 ± 5.0
Kaempferol derivatives	1.57 ± 0.30	1.64 ± 0.24
