# Supplementary figures and images for: Desiccation Tolerance in Ramonda serbica Panc.: An Integrative Transcriptomic, Proteomic, Metabolite and Photosynthetic Study
Source: Plants (Basel). 2022 Apr 28;11(9):1199. doi: 10.3390/plants11091199 (PMC9104375; doi:10.3390/plants11091199)

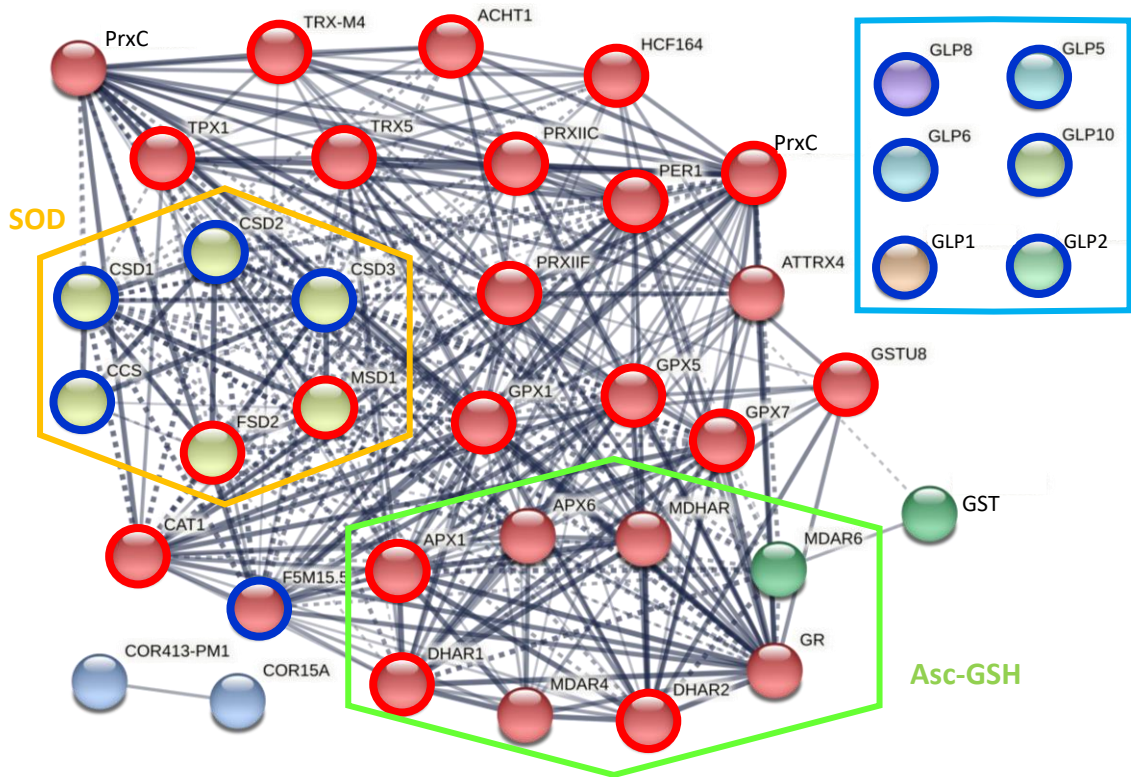

Supplement: Supplementary file 1 [file plants-11-01199-s001.zip › Supplementary Figure S2.pdf]
